# Neuroinflammation in Amyotrophic Lateral Sclerosis and Frontotemporal Dementia and the Interest of Induced Pluripotent Stem Cells to Study Immune Cells Interactions With Neurons

**DOI:** 10.3389/fnmol.2021.767041

**Published:** 2021-12-14

**Authors:** Elise Liu, Léa Karpf, Delphine Bohl

**Affiliations:** Sorbonne Université, Institut du Cerveau – Paris Brain Institute – ICM, INSERM, CNRS, AP-HP, Hôpital de la Pitié-Salpêtrière, Paris, France

**Keywords:** ALS (amyotrophic lateral sclerosis), FTD (frontotemporal dementia), inflammation, immune system, iPSC (induced pluripotent stem cells), immune modulatory molecules

## Abstract

Inflammation is a shared hallmark between amyotrophic lateral sclerosis (ALS) and frontotemporal dementia (FTD). For long, studies were conducted on tissues of post-mortem patients and neuroinflammation was thought to be only bystander result of the disease with the immune system reacting to dying neurons. In the last two decades, thanks to improving technologies, the identification of causal genes and the development of new tools and models, the involvement of inflammation has emerged as a potential driver of the diseases and evolved as a new area of intense research. In this review, we present the current knowledge about neuroinflammation in ALS, ALS-FTD, and FTD patients and animal models and we discuss reasons of failures linked to therapeutic trials with immunomodulator drugs. Then we present the induced pluripotent stem cell (iPSC) technology and its interest as a new tool to have a better immunopathological comprehension of both diseases in a human context. The iPSC technology giving the unique opportunity to study cells across differentiation and maturation times, brings the hope to shed light on the different mechanisms linking neurodegeneration and activation of the immune system. Protocols available to differentiate iPSC into different immune cell types are presented. Finally, we discuss the interest in studying monocultures of iPS-derived immune cells, co-cultures with neurons and 3D cultures with different cell types, as more integrated cellular approaches. The hope is that the future work with human iPS-derived cells helps not only to identify disease-specific defects in the different cell types but also to decipher the synergistic effects between neurons and immune cells. These new cellular tools could help to find new therapeutic approaches for all patients with ALS, ALS-FTD, and FTD.

## Introduction

Inflammation is a pathological hallmark shared by many neurodegenerative diseases. Its physiological function is to defend our organism against various insults implying different cell types and molecular pathways. In many neurodegenerative diseases, insults may come from the different disease-affected cells that can degenerate and die or that can secrete abnormal proteins that become immunogenic. Today, it is well documented that inflammation is not just an inert bystanding secondary reaction. Its modulation could be of interest in a therapeutic perspective, especially for patients with sporadic forms of diseases. Modulating the inflammatory response could then be a strategy to slow disease progression.

This review focuses on amyotrophic lateral sclerosis (ALS) and frontotemporal dementia (FTD), two neurodegenerative diseases with some overlapping clinical presentations, pathological mechanisms, and genetics. Firstly, we will present the current knowledge about inflammation in patients, new hypotheses brought by animal studies and the current state of clinical trials targeting inflammation. Next, we will discuss involvements of both innate and adaptive immune responses and the sequence of inflammatory events. This sequence of events could be a key to identify a time window that could be precisely targeted to twist the immune system in the right direction and slow down disease progression. With a detailed picture of inflammatory events in ALS and FTD, the current possibilities offered by the induced pluripotent stem cell (iPSC) technology to generate different human immune cell types and to study their intrinsic defects will be described. With the emergence of more integrated cellular approaches with different iPS-derived cell types, interactions and synergistic effects between immune cells and neurons could be deciphered and bring new insights for innovative therapeutic approaches for ALS and FTD.

## Amyotrophic Lateral Sclerosis and Frontotemporal Dementia Pathologies

ALS and FTD disorders are two ends of a spectrum of neurodegenerative diseases. ALS clinical presentation is characterized by progressive paralysis of voluntary muscles due to loss of both upper and lower motor neurons (MNs), leading in most cases to the death of patients by respiratory failure. ALS shares clinical and pathological features with FTD, a type of dementia characterized by impaired judgment and executive skills. In FTD, the loss of neurons in the frontal and temporal cortices, sometimes accompanied with a loss of cortical MNs, correlates clinically with the symptoms of FTD (Neumann et al., 2006; Burrell et al., 2016).

Mean survival is 3–5 years for ALS patients and up to 50% of the patients develop frontal lobe dysfunction or language impairment. Among FTD cases, some studies claim only 5–10% of patients with ALS signs (Rosso et al., 2003; Goldman et al., 2005; Johnson et al., 2005; Seelaar et al., 2007), while some others reach 50% (Lipton et al., 2004; Mackenzie and Feldman, 2005). These differences are certainly due to variability in disease definitions and assessments by clinicians. Some more recent studies indicate that FTD-ALS has a particularly poor prognosis with a survival of 2–5 years (Kansal et al., 2016).

At the genetic level, several genes with numerous pathogenic variants, are causal for ALS, ALS-FTD, and FTD (Belzil et al., 2016). The same genetic mutation can result in either ALS, FTD or both pathologies, suggesting roles of disease-specific modifiers. Also, as most mutated genes encode ubiquitously expressed proteins, all cell types can in theory be affected by the expression of the mutated protein, thus contributing to the complexity of the disease (Ferrari et al., 2011). While mutations in the progranulin gene (GRN) and the microtubule-associated protein tau (MAPT) have been identified as major causes of familial FTD (Greaves and Rohrer, 2019), some other genes were linked only to ALS, including kinesin family member 5A (KIF5A) and SOD1 (superoxide dismutase 1). Of particular interest in the ALS-FTD spectrum, mutations in the C9orf72 gene which were identified in 2011, and made the link between both disorders (Ferrari et al., 2019). In the Western hemisphere (and not in Asia), hexanucleotide repeat expansions in the C9orf72 gene have been identified in up to 40% of familial ALS (fALS) patients and 20% of fFTD patients, and in ∼6% of sporadic ALS (sALS) and sFTD patients (DeJesus-Hernandez et al., 2011; Renton et al., 2011). Moreover, recent studies shed light on the role of some genes mutated in both ALS and FTD (TBK1, OPTN, and SQSTM1) and that are implicated in innate immune related functions (Abramzon et al., 2020), warranting the importance of the contribution of immune cells in the pathology.

## Inflammation in Amyotrophic Lateral Sclerosis and Frontotemporal Dementia: A Shared Pathological Hallmark

### First Observations in Post-mortem Tissues of Patients

Post-mortem studies brought the earliest observations suggesting of the presence of inflammatory signs in ALS and FTD patients.

Several ALS post-mortem case reports have described lower numbers of MNs in the spinal cord and to a lower extend of Betz cells in the cerebral cortex, accompanied by increased microglial activation and astrogliosis (Brownell et al., 1970; McGeer et al., 1993; Nihei et al., 1993; Schiffer et al., 1996; Saberi et al., 2015; Chiot et al., 2020). There was no microgliosis in the dorsal horns of the spinal cord, strongly suggesting a specific response of microglial cells toward degenerating MNs. More recent studies identified the presence of immature and activated dendritic cells (DC) in ventral horns and corticospinal tracts of ALS patients (Henkel et al., 2004) as well as the presence of activated CD68+ monocytes/macrophages/microglial cells and of CD4+ and CD8+ lymphocytes in the vicinity of MNs (Kawamata et al., 1992; Henkel et al., 2004). At the periphery, demyelination, axonal degeneration, macrophage activation, abnormal motor end plates, axonal sprouting, and atrophic muscles were described (Bjornskov et al., 1984; Tandan and Bradley, 1985; Chiot et al., 2020).

In FTD patients, inflammatory signs are less obvious compared to ALS patients. Asymmetrical convolutional atrophy in frontal and anterior lobes were observed (Ikeda, 2000; Tolnay and Probst, 2001; Bright et al., 2019). In the gray matter, microvacuolation and gliosis in laminae I–III were seen in conjunction with neuron loss, while neurons of lamina V were reported to be only mildly affected. Rare dystrophic neurites were described. In the white matter, mild gliosis was observed in subcortical fibers and loss of myelin was sometimes observed.

Thanks to these first studies, inflammatory signs were identified in ALS and FTD post-mortem tissues. Nevertheless, it is difficult to define when inflammation begins and if this is an early or late event as post-mortem tissues represent rather an end-stage of the pathology. However, the question of the sequence of the inflammatory events is crucial as inflammation can be beneficial or harmful for neurons depending on the disease stage. Today, this question is an open question that needs more investigation.

### Human Studies to Decipher the Involvement of Inflammation in Amyotrophic Lateral Sclerosis and Frontotemporal Dementia

In humans, studies were conducted at several levels including brain imaging studies and biofluids analysis.

Imaging studies are valuable tools to assess cerebral changes, spreading patterns, network-wise propagations, and can also be used to detect inflammation. These imaging tools could even be used to detect biomarkers to define the conversion transition from a pre-symptomatic stage to a clinically manifest disease (Chew and Atassi, 2019; El Mendili et al., 2019). Positron Emission Tomography (PET) is a functional imaging technique using radioactive ligands to measure, amongst others, changes in metabolic processes. Different radioligands targeting specific cellular substrates are available for different imaging purposes depending on the studied cellular event. TSPO, which corresponds to the 18 kD translocator protein, is highly expressed on activated microglia and astrocytes (Lavisse et al., 2012; Betlazar et al., 2018) and is helpful to visualize inflammation and/or gliosis. Several generations of TSPO radio-ligands exist, which differed regarding their binding specificities. The latest in use are [11C]PBR28 and [18F]DPA-714 which bind with higher specificities to TSPO in comparison to the previous generation of radioligands (Kreisl et al., 2010; Narayanaswami et al., 2018). However, recent studies showed the presence of a polymorphism affecting the TSPO binding affinity, assessing the importance to take into account this parameter in studies involving heterogeneous populations of ALS or FTD patients. A new PET probe [18F]CB251 was recently published and seems to be more specific for TSPO regardless of polymorphisms (Kim et al., 2020). This new probe will be of particular interest for future studies.

The overwhelming majority of imaging studies in patients are done in the brain. Studies in control subjects and ALS patients revealed an increased binding of [18F]DPA-714 (Corcia et al., 2012) or [11C]-PBR28 (Schain and Kreisl, 2017) only to the motor cortex regions with positive correlations with the Upper Motor Neuron Burden Scale (UMNB) and negative ones with the Amyotrophic Lateral Sclerosis Functional Rating Scale – Revised (ALSFRS-R) (Zürcher et al., 2015; Alshikho et al., 2016, 2018; Ratai et al., 2018). In FTD and ALS-FTD patients, increased TSPO binding were observed in cortical frontal, mesial temporal, subcortical regions, prefrontal cortex, hippocampal, and para-hippocampal regions (Cagnin et al., 2004; Turner et al., 2004; Miyoshi et al., 2010; Chew and Atassi, 2019).

Despite numerous attempts to image the spinal cord *in vivo* (Bede et al., 2012) technological constraints (i.e., respiration, cardiac movements, and small cross-sectional area) have hindered reliable quantitative spinal cord imaging in patients (El Mendili et al., 2019). Amongst the scarce imaging studies assessing ALS patient’s spinal cords, most of them used magnetic resonance imaging (MRI) techniques. Studies of metabolic changes in ALS patients’ spinal cords using whole body PET/computed tomography (CT) images are recent. Two independent studies identified increased [18F]-fluorodeoxyglucose (FDG) uptake in the spinal cord of patients. [18F]-FDG is thought to reflect cell metabolism without specificity for glial cells, but the observed hyper-metabolism in the spinal cord was suggested to represent an increased inflammation and gliosis due to accumulating glial cells in reaction to degenerating neuronal cells (Bauckneht et al., 2020). Further studies using specific radioligands will have to confirm these observations.

PET studies opened new perspectives regarding our understanding of pathology and inflammation in patients. To go a step further, it might now be possible to assess temporal changes during the disease course. Objectives are to observe cell reactivity as well as spreading of inflammation and gliosis in ALS and FTD patients’ brains and spinal cords. In the largest longitudinal ALS PET study, 10 patients underwent [11C]-PBR28 PET scans twice over a 6 months period. Results showed a stable [11C]-PBR28 uptake over this period of time (Alshikho et al., 2018), suggesting a plateau of glial reactivity shortly after symptoms onset. These first results are very encouraging suggesting that the inflammation does not increase with time. Other longitudinal studies are now necessary to support these results. If asymptomatic subjects of patient’s families could be included in these studies, this could help understand the crucial question of when the neuroinflammatory response starts.

As a whole, imaging studies are particularly interesting as they offer insights in the status of brain and spinal cord pathological tissues at spatial and temporal levels. They also bear hope as a tool to detect biomarkers. Nevertheless, imaging studies still hold limitations for studying precisely microglial activation in ALS and FTD patients. As mentioned previously, TSPO is not highly cell-specific (Lavisse et al., 2012), which limits data interpretations (Vivash and O’Brien, 2016). Thus, other radioligands targeting microglia more specifically are currently developed and first studies have already shown PET imaging with a tracer targeting the pro-inflammatory phenotype of activated microglial (Janssen et al., 2018; Narayanaswami et al., 2018). The next step is now to develop a tracer for the anti-inflammatory phenotype of microglia. This would allow for imaging of the different microglia activation states during disease evolution.

### Circulating Inflammatory Cytokines and Chemokines in Amyotrophic Lateral Sclerosis and Frontotemporal Dementia

The presence of circulating inflammatory cytokines and chemokines in the blood and the cerebrospinal fluid (CSF) was extensively studied in ALS patients and to a lesser extent in FTD patients. Recently, studies showed that the pro-inflammatory and multifunctional IL-6 cytokine was increased in both ALS and FTD patients in comparison to controls (Galimberti et al., 2015; Gibbons et al., 2015; Ngo et al., 2015; Lu et al., 2016; Tortelli et al., 2020). Furthermore, the IL-6 level was suggested to be correlated with disease progression in ALS (Lu et al., 2016). Apart from IL-6, ALS and FTD patients have distinct cytokines and chemokines circulatory profiles (see below and Table 1 that presents the different publications studying circulating inflammatory molecules in CSF and blood of ALS or FTD patients).

**TABLE 1:** Publications reporting CSF and blood circulating inflammatory molecules in ALS and FTD patients.

Compartment	ALS	FTD
CNS	Increased circulating factors in CSF: cytokines and chemokines: IL-2, IL-4, IL-5, IL-7, IL-8, IL-9, IL-12, IL-15, IL-17, IFN-γ, TNF-α, eotaxin, CCL11, MIP-1α, MIP-1β, MCP-1, IP-10 (Tanaka et al., 2006; Kuhle et al., 2009; Mitchell et al., 2009; Tateishi et al., 2010; Gonzalez-Garza et al., 2018); growth factors: FGF-2, VEGF, G-CSF, GM-CSF, PDGF-BB (Tanaka et al., 2006; Mitchell et al., 2009; Tateishi et al., 2010; Furukawa et al., 2015; Guo et al., 2017) Contradictory results: increase or decrease of IL-10 (Tanaka et al., 2006; Mitchell et al., 2009)	Increased circulating cytokines in the CSF: IL-8, IL-11, IL-23, MCP-1, IP-10, Eotaxin-3, TGF-β1, YKL40 (Sjögren et al., 2004; Galimberti et al., 2006, 2008, 2015; Hu et al., 2010; Bossù et al., 2011; Teunissen et al., 2016) Decreased circulating cytokines in the CSF: IL-12, IL-15, IL-17, TNF-α, RANTES (Rentzos et al., 2006a; Hu et al., 2010; Galimberti et al., 2015) Increased inflammatory lipids in the CSF: LPC, PAF (Phan et al., 2020)
Blood	Increased circulating factors linked with extracellular matrix remodeling: MMP9, TIMP2 (Andrés-Benito et al., 2017); oxidative stress markers: GSSG, 8-OHdG, MDA (Blasco et al., 2017); circulating cytokines/chemokines: IL-1β, IL-4, IL-8, IL-12p70, IL-13, IL-15, IL-17A, IL-18, TNF-α, MIP-1α, MCP-1, eotaxin (Kuhle et al., 2009; Italiani et al., 2014; Ehrhart et al., 2015; Ngo et al., 2015; Lu et al., 2016; Blasco et al., 2017; Guo et al., 2017; Prado et al., 2018; Tortelli et al., 2020; Brodovitch et al., 2021); growth factors: G-CSF, GM-CSF, bFGF, VEGF (Guo et al., 2017) Downregulation of inflammatory cytokines chemokines CCL5, CXC5R, TGF-β2, IL-10RA (Ehrhart et al., 2015; Andrés-Benito et al., 2017), oxidative stress markers: GSH (Ehrhart et al., 2015; Blasco et al., 2017) Contradictory results: increase or decrease of IL-2, IL-5, IL-6, IL-10, IFN-γ (Ehrhart et al., 2015; Lu et al., 2016; Andrés-Benito et al., 2017; Blasco et al., 2017; Guo et al., 2017; Prado et al., 2018; Tortelli et al., 2020; Brodovitch et al., 2021)	Increased peripheral circulating cytokines IL-6, IL-8, IL-15, IL-17, CCL26, MCP-1, IP-10, TNF, FasL, TRAILR3 (Bossù et al., 2011; Miller et al., 2013; Gibbons et al., 2015) Decreased peripheral circulating cytokines IL-1α, IL-6, IL-12, IL-23, RANTES, TNF (Santos et al., 2014; Galimberti et al., 2015)

*This table recapitulates circulating inflammatory molecules measured in the CSF and the blood of patients. The molecules can be secreted by several immune cell types. 8-OHdG, 8-hydroxydesoxyguanosine; bFGF, basic fibroblast growth factor; CCL5, C-C motif chemokine ligand 3; CCL11, C-C motif chemokine ligand 11; CCL26, C-C motif chemokine ligand 26; CXC5R, C-X-C motif chemokine receptor 5; FasL, Fas ligand; FGF-2, fibroblast growth factor 2; G-CSF, granulocyte-colony stimulating factor; GM-CSF, granulocyte-macrophage colony-stimulating factor; GSH, glutathione; GSSG, glutathione disulfure; IFN-γ, interferon-gamma; IL-1β, interleukin-1 beta; IL-2, interleukin-2; IL-4, interleukin-4; IL-5, interleukin-5; IL-6, interleukin-6; IL-7, interleukin-7; IL-8, interleukin-8; IL-9, interleukin-9; IL-10, interleukin-10; IL-10RA, interleukin 10 receptor subunit alpha; IL-11, inteleukin-11; IL-12, interleukin-12; IL-12p70, interleukin-12p70; IL-13, interleukin-13; IL-15, interleukin-15; IL-17, interleukin-17; IL-17A, interleukin-17A; IL-18, interleukin-18; IL-23, interleukin-23; IP-10, interferon gamma-induced protein 10; LPC, lysophosphatidylcholine; MCP-1, monocyte chemoattractant protein 1; MDA, malondialdehyde; MIP-1α, macrophage inflammatory protein-1 alpha; MIP-1β, macrophage inflammatory protein-1 beta; MMP9, matrix metallopeptidase 9; PAF, platelet-activating factor; PDGF-BB, platelet-derived growth factor-BB; RANTES, Regulated Upon Activation, Normally T-Expressed, and Presumably Secreted (or CCL5); TGF-β1, transforming growth factor beta 1; TGF-β2, transforming growth factor beta 2; TIMP2, tissue inhibitor of metalloproteinases 2; TNF-α, tumor necrosis factor alpha; TRAILR3, tumor-necrosis-factor related apoptosis inducing ligand receptor 3; VEGF, vascular endothelial growth factor; YKL-40, chitinase 3-like 1.*

In ALS, a large panel of pro and anti-inflammatory cytokines and chemokines were described as deregulated in patients (reviewed in Moreno-Martinez et al., 2019). However, results are not always consistent amongst the different studies, probably because of the heterogeneity of the studied populations. Heterogeneity exists at different levels. First, in most reports the studied population has a mean ALSFRS-R with a large standard deviation and patient’s biofluids are analyzed at one single time point. Thus the inflammation status of the different patients may be different, making the results hard to interpret. Of note, the disease state is most of the time measured with the ALSFRS-R, but it does not necessarily reflect the pathological state at the cellular level. To date among the most promising biomarkers for ALS are neurofilaments heavy and light chains, measurable in CSF and blood and being used in many trials (Oeckl et al., 2016; Xu et al., 2017; Benatar et al., 2018; Poesen and Van Damme, 2019). Second, some studies include patients diagnosed with the El Escorial criteria that categorize the disease as “possible,” “probable,” or “definite.” This classification could allow establishing if there is a correlation between the diagnosis categories and the amount of circulating factors. However, some other studies take all diagnostic cases as one single group of patients and do not take into account the diagnosis, making any correlation impossible. Third, the site of onset of the pathology (spinal vs bulbar) may also be a confounding factor if the two groups are not considered separately. Indeed, progression of the disease is very different between the two forms and so are probably the inflammatory events over time. Next, most studies include sporadic cases or compare familial and sporadic cases, which cannot be seen anymore as different groups. Better defining patient groups are needed. As we now know the importance of some mutated genes in the immune system, retrospective studies could be interesting to compare sub-groups based on their genotypes. (v) Finally, CSF and blood are distinct compartments as the CSF is part of the central nervous system (CNS) while circulating peripheral molecules are in the blood. Both compartments may contain different types and amounts of cytokines and chemokines depending on the state of the pathology.

Few longitudinal studies were conducted and no significant differences in cytokines and chemokines secretions were observed between two pathological time-points in patients (Saleh et al., 2009; Ehrhart et al., 2015; Prado et al., 2018). As in most cases recruited patients were already in advanced stages of the disease, a hypothesis is that if inflammation is an early event, analysis was probably conducted too late. Having the opportunity to do this kind of analysis as early as possible after diagnosis could bring new insights of the inflammatory appearance and evolution with disease progression.

Amongst other factors measured in ALS patients’ biofluids, immunoglobulins (IgG), metabolic proteins, adipokines, iron-related proteins, and oxidative stress markers were found deregulated in patients (Mitchell et al., 2010; Ngo et al., 2015; Blasco et al., 2017). These deregulations reflect metabolic and inflammatory dysfunctions which may be critical actors of pathogenesis. In vitro and *in vivo* studies demonstrated that IgG of ALS patients induced a selective MN’s death, presumably involving calcium (Yi et al., 2000; Obal et al., 2002; Pullen et al., 2004; Demestre et al., 2005), supporting a direct role of immunological responses on the selective MN loss.

In FTD patients, most studies focused on CSF analysis. Analyses revealed altered secretions of inflammatory chemokines and cytokines as those found in ALS patients, such as IP-10, TNFα (Sjögren et al., 2004), TGFβ, CCL-2 (also called MCP-1) (Galimberti et al., 2015), RANTES (Galimberti et al., 2008), and IL-8 (Galimberti et al., 2006). Some cytokines seemed more specific to cognitive aspects of the pathology as they were found deregulated in Alzheimer’s disease (AD) and FTD patients such as IL-11 (Galimberti et al., 2008), IL-15 (Rentzos et al., 2006b), and IL-12 (Rentzos et al., 2006a). Interestingly, in clinical cases with FTLD-TDP and FTLD-tau (2 different FTD forms) pathologies, differences in some neuropeptides and chemokines secretions (IL-23 and IL-17) were observed, a signature that could help to distinguish the two forms when antemortem follow-ups are done (Hu et al., 2010). Regarding blood analysis, no statistical differences of FTD patients’ serum cytokines compared to controls were found (Galimberti et al., 2015). On the other hand, lipid assays showed increase triglyceride levels in patients correlated with body mass index, while HDL cholesterol levels were negatively correlated with this index (Ahmed et al., 2014). More recently a serum lipidomic study focused more precisely on lipids implied in 3 key aspects of FTD pathology: inflammatory processes, mitochondrial dysfunction, and oxidative stress (Phan et al., 2020). Amongst other findings, the data revealed specific increased lipid implicated in inflammatory responses, LPC and PAF (lysophosphatidylcholine and platelet-activating factor) which are known as second messengers or immediate response molecules and which act on various immune targets (T lymphocytes, microglia, macrophages, and neutrophils) (Chang et al., 2003; Han et al., 2004; Scholz and Eder, 2017). In contrast, they found a decrease in o-acyl-w-hydroxy fatty acids (OAHFA) which is known to exert anti-inflammatory effects. The significant inverse correlation between LPC and OAHFA variations suggests increased inflammation in FTD patients.

Taken together, these studies show that inflammatory events occur in ALS and FTD patients. What is now needed is a better understanding of the actual start and evolution of these inflammatory events in both pathologies, which would be important for targeted immunomodulating therapies.

## The Role of the Immune System in Amyotrophic Lateral Sclerosis and Frontotemporal Dementia

**FIGURE 1:**
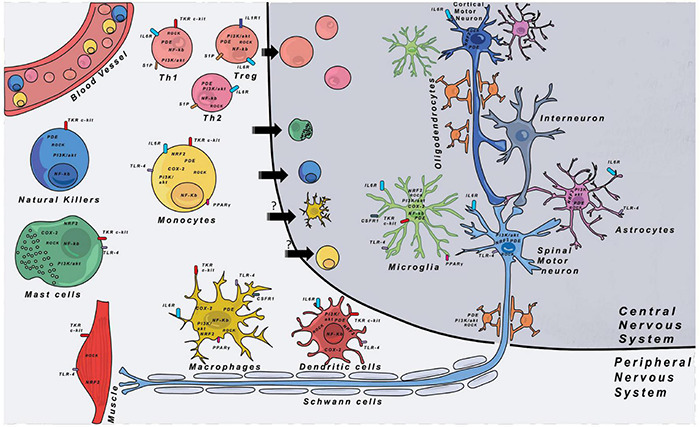
Immune cells and their respective locations in the CNS and PNS. Major therapeutic targets are shown and are located either on the cell membrane or in the cytoplasm of cells. The expression of the different targets was indexed through researches with proteinatlas.org. COX-2, cyclooxygenase-2; CSF1R, colony stimulating factor 1 receptor; IL-1R1, interleukin-1 receptor 1; IL-6R, interleukin-6 receptor; NF-κB, nuclear factor kappa-light-chain-enhancer of activated B cells; NRF2, nuclear factor erythroïd-2-related factor 2; PDE, phosphodiesterase; PGE2, prostaglandin E2; PI3K, phosphoinositide 3-kinase; PPARγ, peroxisome proliferator-activated receptor gamma; ROCK, Rho-associated protein kinase; S1P, sphingosine-1-phosphate receptor; TKR, tyrosine-kinase receptor; TLR-4, Toll-like receptor 4.

Inflammation is a multifaceted reaction implying different cell types and molecular pathways. Figure 1 shows the different immune cells that can play roles during disease progression both in the CNS and the PNS. Below we describe the different cell types that are thought to play a role in inflammatory mechanisms in ALS and FTD, and how they might impact the disease progression.

### Innate Immunity in Amyotrophic Lateral Sclerosis and Frontotemporal Dementia

Innate immunity is the first line defense of the organism. It provides an immediate immune response against a non-self pathogen and is conserved amongst vertebrates and invertebrates. It encompasses a group of cell types that recognize attacks on the organism and facilitate the clearance of external pathogens. These cell types have specific functions depending on their tissue location and surrounding environment. Table 3 lists published data indicating the proposed implications of the different immune cell types both in animal models of ALS and FTD and in patients.

#### Phagocytes

Amongst phagocytes different subpopulations exist. Tissue resident macrophages are heterogeneous populations that can differ by their embryonic origin but also their tissue location where they acquire specific functions and distinct profiles.

Macrophages are antigen presenting cells (APC). The main role of APC is to process antigens and present them by major histocompatibility complex (MHC) at cell surface. The MHC-antigen complex is recognized by the T cell receptor (TCR), allowing T cell activation. APC also express many co-stimulatory molecules that participate in T cell activation. APC regroup several immune cell types in different tissues, including DC, macrophages, B cells and Langerhans cells (Kambayashi and Laufer, 2014). Macrophages are less potent than DC but still enough to activate neighboring adaptive immune cells. Macrophage activation states are multiple (Xue et al., 2014). Depending on the insult they encounter, they can release different cytokines, chemokines, and other factors to drive inflammation by activating neighboring cells but also attracting blood immune cells to the site of injury. At the same time, their key phagocytic activities allow them to clean up cellular debris and thus decrease local inflammation. Microglia are the CNS resident macrophages but have a distinct embryonic origin compared to the majority of peripheral macrophages (Ginhoux et al., 2010; Schulz et al., 2012; Kierdorf et al., 2013; Gomez Perdiguero et al., 2015). Since decades, the understanding of the exact roles of microglia in the CNS is a field of intense research. These cells display extended prolongations allowing them to patrol the whole brain parenchyma within a matter of hours, making them very active cells in the CNS. While microglia have been long studied for their roles during brain development, their homeostatic roles in the adult are less clearly defined. However, during neuronal injury or neurodegeneration, they function as macrophages and appear to be the first line of response to neuronal suffering.

Today, accumulating evidence points to important roles of microglia in ALS and FTD. However, for a long time, the lack of specific marker to distinguish peripheral macrophages (able to invade the CNS) from microglia, made it difficult to study the two cell populations individually. Thus, especially for *in vivo* studies in ALS rodent models, often, both cell types were studied as a single population. In 2006, two independent studies demonstrated that decreasing expression of mutant human SOD1 (hSOD1) only in macrophages/microglia in a mutant hSOD1 ALS mouse model led to slowing of disease progression and increased survival (Beers et al., 2006; Boillée et al., 2006). In ALS, the degenerating spinal MN projects its longest part, the axon, into the periphery, and thus, the axon would be in contact with reacting macrophages and other peripheral immune cells, while the soma in the CNS would be in contact with microglia.

This particularity of the spinal MN led to the idea that in ALS it might be a promising approach to target directly the peripheral immune system and the macrophages along the peripheral motor nerves to slow neurodegeneration. This could be potentially an easier therapeutic approach than targeting microglia in the CNS, and by targeting neuroinflammation could be used for all ALS cases. This idea was applied in a recent article that demonstrated that gene expression patterns were very different during the disease in microglia and macrophages in mutant hSOD1 mice (Chiot et al., 2020) showing that the two cell types can play different roles in the disease. In this study, replacement of peripheral mutant SOD1 macrophages by more neurotrophic macrophages decreased both sciatic nerve macrophage activation and CNS microglial activation (Chiot et al., 2020). This is particularly interesting as it shows that by modulating macrophages at the periphery it is possible to act on CNS inflammation. Interestingly, Chiot et al. (2020) demonstrated that when this replacement was done at a pre-symptomatic stage, disease onset was delayed, but this was not sufficient to increase life expectancy, whereas when the macrophage replacement was done at disease onset, it was able to increase survival of ALS mice. The necessity of a precise timing for the macrophage replacement in this ALS model could give insights into the time window that has to be targeted to act on peripheral inflammation in ALS. Whereas most of the studies regarding the roles of phagocytes were done with the mutant SOD1 mouse model, in which phagocytes showed deleterious effects on disease progression, a recent study suggested potential neuroprotective effects of reactive microglia in one TDP-43 mouse model (Spiller et al., 2018). These results show that the involvement of phagocytes is context-dependent and thus has to be studied in several ALS models and in humans with different disease forms to understand their exact roles during the disease course.

In FTD and ALS, two of the most commonly found mutant genes (GRN, C9orf72) encode proteins that have critical roles in phagocytosis and endocytosis which are important for microglial functions (Baker et al., 2006; Cruts et al., 2006; Shatunov et al., 2010; DeJesus-Hernandez et al., 2011; Mok et al., 2012). Progranulin acts as an inflammatory modulator and its expression is significantly up-regulated in microglia in several models of neuronal injury (Moisse et al., 2009; Naphade et al., 2010; Tanaka et al., 2013). In GRN knockout mice aberrant microglial activation upon stimulation and during aging was reported (Yin et al., 2009; Martens et al., 2012; Lui et al., 2016), leading to increased synaptic pruning or even neuronal loss. GRN deficient mice harbor obsessive-compulsive behaviors which seem to imply the TNFα signaling pathway predominant in microglial cells in the CNS (Krabbe et al., 2017). This major immune reaction can be alleviated through restoration of progranulin level. Indeed, an overexpression of the latter in a mouse model of sciatic nerve injury led to accelerated axonal regrowth, restoration of neuro-muscular junctions, and recovery of sensory and motor functions (Altmann et al., 2016). Regarding C9orf72 studies, its deficiency in an animal model showed a hyper activation of the innate immune system with increased expression of IL-6 and IL-1β in microglia and upregulated inflammatory genes in the spinal cord (ORourke et al., 2016). Moreover, several studies reported a defective lysosomal system with important accumulations in innate immune cells (Atanasio et al., 2016; Sullivan et al., 2016; Moens et al., 2017). Together these results show a prominent role of C9orf72 in microglial cells. The phagocytic machinery is essential for clearance of cell debris and maintenance of homeostasis. Disturbance in the correct functioning of this machinery could lead to aberrant neuroinflammation and thus could directly contribute to the disease process in ALS and FTD rather than being a simple secondary event to neuronal degeneration. Nonetheless, these results need to be interpreted cautiously as both models do not match haploinsufficiency found in ALS and FTD patients.

Aside from these studies in animal models, most human studies have used circulating blood monocytes, as access to tissue resident macrophages and microglial cells are unlikely possible in live patients.

In ALS patients, different studies have assessed peripheral monocyte populations. An increase of classical and a decrease of non-classical monocytes was observed not only in sALS and fALS patients, in line with findings in the mutant hSOD1 mice, but also in pre-symptomatic ALS mutant carriers (Butovsky et al., 2012; Zondler et al., 2016), suggesting that this phenotype is an early event in the pathology. Moreover, circulating monocytes of ALS patients presented functional defects in phagocytosis and migration, and appeared to be skewed toward a more pro-inflammatory phenotype with a TNFα protein release positively correlating with progression rates, and a IL-6 protein release positively correlating with disease burden (Zondler et al., 2016; Zhao et al., 2017; Du et al., 2020). Deep RNA sequencing of ALS monocytes revealed unique inflammation-related gene profiles, defects in migration and in the lysosomal pathway (Zondler et al., 2016; Zhao et al., 2017). While aforementioned studies were carried out on blood monocytes, a recent study observed that ALS monocytes-derived macrophages had increased IL-6 and TNFα secretion levels when activated toward a pro-inflammatory state, suggesting that blood monocyte-derived macrophages kept at least some of their *in vivo* characteristic defects when in culture (Du et al., 2020). Other studies are now needed to better understand the inflammatory states of ALS macrophages at different time-points of the pathology, and this could be assessed by using monocytes, easy accessible from alive patients.

In FTD patients, a study revealed increased monocytes in the CSF of patients with no change in the proportion of non-classical compared to classical monocytes (Pawlowski et al., 2018). Elevated monocyte levels correlated with structural cerebral defects in FTD typical affected regions assessed by structural MRI (Pawlowski et al., 2018). Further studies would be important to validate these alterations at a larger scale.

#### Dendritic Cells

Dendritic cells orchestrate innate and adaptive complex systems. DC are the most efficient APC to activate T cells into specific lineages because they display more MHC classes and co-stimulatory molecules compared to others APC (Inaba et al., 1997). In the periphery, immature DC patrol to find non-self antigens throughout tissues. The encounter with a pathogen or a danger signal triggers their maturation, antigen presentation on MHC-II and migration to secondary lymphoid organs where they activate CD4 or CD8 T cells. At steady-state level, DC can present self-antigens to T cells to avoid immune responses against self-proteins and induce tolerance. This process leads to the induction of regulatory T cells (Tregs) that express and secrete anti-inflammatory molecules to reduce inflammation (Banchereau and Steinman, 1998).

In sALS and fALS patient’s post-mortem tissues, immature and mature DC were predominantly observed in the degenerating corticospinal tracts, with increased mRNAs levels coding for DC surface markers and for the inflammatory chemokine CCL-2 (Henkel et al., 2004). Interestingly, patients with a rapid disease progression had more transcripts compared to patients with a slower progression. This suggests an active recruitment of DC by the inflamed CNS of ALS patients. A hypothesis is that besides the known CCL-2 sources (principally attributed to macrophage and microglia) and with a role of CCL-2 as a chemoattractant for monocytes and T cells, DC could be an additional CCL-2 source, therefore participating in inflammatory cell recruitment. In agreement with this hypothesis is that several studies described an increase of CCL-2 in ALS patient’s CSF (Baron et al., 2005; Kuhle et al., 2009; Tateishi et al., 2010; Gupta et al., 2011). Also in the blood of ALS patients, a subpopulation of circulating DC showed an increased production of CCL-2 in response to LPS (Rusconi et al., 2017).

Further analysis of circulating DC in blood of ALS and FTD patients would be of great interest if the different subpopulations of DC could be identified. cDC1 have the ability to highly cross-present antigen to CD8 T cells, while cDC2 is the most efficient DC subset to polarize naive CD4 T cells (Haniffa et al., 2013). pDC are a main actor of antiviral responses by the production of type 1 interferon (IFN-1) (Dzionek et al., 2000). Finally, it was shown that monocyte-derived DC (MoDC) can arise during inflammation. MoDC have been first observed in mice (León et al., 2007) and then in humans under inflammatory physiological and physiopathological conditions (Segura et al., 2013; Tang-Huau et al., 2018).

#### Mast Cells

Mast cells are long-lived tissue resident cells, implicated in many different inflammatory responses. As they encounter specific antigens, they activate and release numerous inflammatory mediators (i.e., histamine, cytokines, lysosomal enzymes, ATP, and serine proteases). They are generally located near structures mediating visceral sensory or neuroendocrine functions or close to the blood-brain-barrier (BBB). In the spinal cord under normal conditions, mast cells are present at the dura but not in the cord parenchyma (Michaloudi et al., 2008). Mast cells are also first line effectors through which pathogens can affect the gut-brain axis (Budzyński and Kłopocka, 2014; Conte et al., 2020).

In the past years, mast cells received increased attention, as they appeared to be an early responder to injury (Skaper et al., 2014). Studies have shown that when they are activated they are important mediators of the microglial inflammatory response, astrocyte activation and potential neuronal degeneration (Skaper et al., 2014; Zhang et al., 2016; Jones et al., 2019). They are also able to disrupt and permeabilize the BBB leading to toxins and immune cells penetration, exacerbating the inflammatory response.

In post-mortem spinal cords of ALS patients, increased Cox-2 mast cells – Cox-2 is a key mediator of the inflammation (Chen, 2010) – were detected while they were not present in controls (Graves et al., 2004), Recently, mast cells were described near the altered microvascular elements and surrounding MN cell bodies (Kovacs et al., 2021). In the mutant hSOD1 rat model, accumulation of mast cells was observed in ventral roots and spinal cords (Trias et al., 2018). Interestingly, mast cells were also found in the periphery and in particular in muscles, with infiltrations and degranulations correlating with paralysis progression (Trias et al., 2017). Mast cells are thought to be recruited along the degenerating nerve by the Stem Cell Factor (SCF) secreted from reactive Schwann cells and reactive macrophages (Trias et al., 2019). Very recently, a report showed that mast cells were interacting with astrocytes and MNs expressing SCF in the mutant hSOD1 mouse model (Kovacs et al., 2021). Pharmacological inhibition of CSF-1R and c-kit with Masitinib [a tyrosine kinase inhibitor targeting the SCF receptor (c-kit) and the platelet derived growth factor (PDGF)] showed reduced immune cell infiltration and amelioration of neuromuscular junction (NMJ) integrity, suggesting an implication of mast cells in the axonopathy in periphery in the ALS pathology (Trias et al., 2016, 2020). Based on these encouraging results suggesting that mast cells participate to inflammatory reactions in ALS both in the CNS and PNS, clinical trials were launched with Masitinib (ongoing trials from AB Science). Whether mast cells are a driver or an amplifier of the ALS pathology remains to be determined. Their “early” implication in the pathology is suggested as at the symptomatic phase in mutant hSOD1 rats, mast cells have already increased in number. Assessing their presence at the pre-symptomatic stage would be of great interest to assess their earlier involvement.

In FTD patients, nothing is known about mast cells participation, but it would not be aberrant to see their increased reactivity as was observed in AD patients (Harcha et al., 2021).

#### Natural Killer Cells

Natural killer (NK) cells are classified as innate cytotoxic lymphocytes and are mainly known for their ability to kill virus-infected cells and tumor cells (Abel et al., 2018). Few papers reported an increase of NK cells in ALS patients’ blood (Gustafson et al., 2017; Jin et al., 2020). Very recently, Garofalo et al. (2020) described an infiltration of p46-positive NK cells in the spinal cord and motor cortex of patients with sALS. They also showed NK cell recruitment in the CNS of SOD1*^G93A^* mice. CCL-2 was shown to be involved directly in it or through other cell recruitment, since CCL-2 neutralization led to a decrease of NK cell infiltration. Interestingly, these data identified CCL-2 as an early damage signal of neural tissue. The depletion of NK cells in SOD1*^G93A^* mice, but also in TDP43*^A315T^* mice, increased survival and delayed paralysis onset. Functionally, control and SOD1*^G93A^* NK cells had a cytotoxic effect on SOD1*^G93A^* MNs, but not on control MNs, suggesting a kill-me signal coming from mutant MNs. Regarding the impact on other immune cells, they showed that NK-depleted SOD1*^G93A^* mice had a decreased number of microglia in spinal cord ventral horns, with a less inflammatory profile. NK depletion also induced an increase of Tregs (see below) in the ventral horns (Garofalo et al., 2020). More studies deserve now to be done to better understand the involvement of NK cells in ALS.

In FTD patients, one study showed that NK cell percentages were not modified in patients’ blood samples (Busse et al., 2017).

Altogether, these data support the critical role of innate immunity in ALS-associated neurodegeneration and to a lesser extend in FTD. Phagocytes, mast cells, DC, and NK cells are activated in early disease phases and thus may participate through positive or negative impacts on neuron degeneration.

### Adaptive Immunity in Amyotrophic Lateral Sclerosis and Frontotemporal Dementia

The adaptive immune system, unlike innate immunity, is highly specific to the particular pathogen that induces it and can provide long lasting immune protection. This type of immunity is strictly confined to vertebrates as it arises in evolution less than 500 million years ago (Alberts et al., 2002). Amongst the cells that constitute this second line of immunity, major actors are T and B cells that can be subdivided in many subclasses that can play different inflammatory roles and serve different purposes during inflammatory events.

#### T Lymphocytes

The T lymphocyte population is composed of two main subpopulations: CD4 and CD8 T cells. They are both characterized by the expression of CD3 and T-cell receptor (TCR) at their membrane surface. During T cell activation, the TCR recognizes antigen peptides presented by APC on MHC-II for CD4 T cells and MHC-I for CD8 T cells. In addition to this TCR/MHC/peptide complex signal, co-stimulatory molecules interactions (the main one being CD28 binding to CD80/CD86) and cytokines are necessary for T cell activation (Kapsenberg, 2003).

##### CD4 T Cells

CD4 T cells, also called T helper (Th) cells, help to set up an appropriate immune response against the encounter pathogen. To this aim, they provide signals to other immune cells that influence their activation and thus guide the immune response according to the pathogen to target. Naive Th cells are activated and polarized by APC, mainly DC, in secondary lymphoid organs. They are classified in several subsets, the main ones being Th1, Th2, Th17, and Treg cells. They are primarily defined by their transcription factor patterns of expressions and their cytokine productions (Table 2).

**TABLE 2:** CD4 T helper cell subsets, their molecular signatures, and their main effector functions in immunity.

CD4 T subsets	Master transcription factors	Secreted cytokines	Effector functions in adaptive immunity
Th1	T-bet	**IFN-γ**	Clearance of intracellular pathogens (e.g., viruses) by cell mediated immunity such as CD8 T cell and macrophage activation (Mosmann and Coffman, 1989)
	STAT4	IL-2 TNF-α	
	STAT1	TNF-β	
Th2	GATA-3	**IL-4**	Clearance of extracellular pathogens and activation of antibody-producing B cells (humoral immunity) (Zhu et al., 2006)
	STAT6	**IL-5**	
		IL-9	
		**IL-13**	
		IL-31	
Th17	RORγt	**IL-17A**	Clearance of extracellular pathogens (mainly at mucosal and epithelial surfaces) Antimicrobial properties against (Liang et al., 2006) Recruitment of neutrophils (Laan et al., 1999)
	RORa	**IL-17F**	
		IL-21	
		IL-22	
Treg	FoxP3	**IL-10**	Immune tolerance Anti-inflammatory function (von Boehmer, 2005)
		**TGF-β**	

*For each CD4 T subset, master transcription factors are indicated as well as their cytokine secretory profile. In bold is indicated the most representative cytokine secreted by the cell subset. FoxP3, forkhead box P3; GATA-3, GATA binding protein 3; IFN-γ, interferon-gamma; IL-2, interleukin-2; IL-4, interleukin-4; IL-5, interleukin-5; IL-9, interleukin-9; IL-10, interleukin-10; IL-13, interleukin-13; IL-17A, interleukin-17A; IL-17F, interleukin-17F; IL-21, interleukin-21; IL-22, interleukin-22; IL-31, interleukin-31; RORa, retinoic acid-related orphan receptor a; RORγt, retinoic acid-related orphan receptor gamma t; STAT1, signal transducer and activator of transcription 1; STAT4, signal transducer and activator of transcription 4; STAT6, signal transducer and activator of transcription 6; T-bet, T-box expressed in T cells; TGF-β, transforming growth factor beta; Th, T helper cell; TNF-α, tumor necrosis factor alpha; TNF-β, tumor necrosis factor beta.*

In ALS, Th cells are described as major players in inflammation and disease progression. In the 1990s, CD4 T cell infiltrations were observed in spinal cords of ALS patients in proximity to degenerating areas (Kawamata et al., 1992; Engelhardt et al., 1993). At this time, the different Th cell subsets had not been all discovered. More recent studies on patient’s blood samples and on some patient’s CNS tissues focused more precisely on the different Th subpopulations and amongst the most deregulated subpopulations, Treg cells received a lot of attention. These cells were found significantly reduced in ALS patient’s blood compared to controls (Mantovani et al., 2009; Henkel et al., 2013; Saresella et al., 2013; Sheean et al., 2018; Jin et al., 2020). In their publication describing significant decreases of FoxP3 mRNAs in circulating leukocytes of ALS patients, Henkel et al. (2013) showed that low FoxP3 mRNA levels correlated with a rapid progression of the disease and a poor survival of patients. Interestingly, similar results were observed in the SOD1*^G93A^* mouse model with decreased Treg cell numbers and increased ones of other Th subsets along disease progression (Beers et al., 2011; Zhao et al., 2012). Recently, Beers et al. (2017) went one step further focusing on the impact of the disease on Treg functions. Treg are known to suppress both innate and adaptive immune reactions detrimental to the host and in particular they can suppress the activation/expansion of neurotoxic T lymphocytes. They demonstrated that Treg isolated from blood of ALS patients were less effective in their capacity to suppress proliferation of responder T lymphocytes. In addition, Treg cells of rapid progression patients (1–2 years, disease duration) exhibited an even more reduced suppression capacity compared to Treg cells of slow progression patients (4–6 years, disease duration). Thus, Treg cell suppressive capacity was inversely correlated with the disease progression speed (Beers et al., 2017). All these results strongly suggesting a direct role of Treg in ALS led to the development of therapeutic proposals to induce Treg production in patients (see below).

Th1 (or IFN-γ-producing CD4 T cells) and Th17 (or IL-17-producing CD4 T cells) cells are increasingly considered as playing key roles in ALS inflammation. In most studies, these two Th subsets were usually studied together because of their pro-inflammatory properties, despite their distinct immunological functions. Th17 cells are already known to be pathogenic in inflammatory diseases such as multiple sclerosis or inflammatory bowel disease (Wu and Wan, 2020). In SOD1*^G93A^* mice abnormal CD4 T cell activation and abnormal Th17 cell numbers were described in the draining cervical lymph nodes prior to the onset of neurological symptoms (Ni et al., 2016). In ALS patient’s blood, both Th1 and Th17 cells were shown to increase (Saresella et al., 2013; Jin et al., 2020) and IL-17 was measured in higher concentration (Rentzos et al., 2010). At the level of the CNS, Henkel et al. (2013) showed an increase of Th1 mRNA markers in the spinal cord of ALS patients. Interestingly, they described an increase of T-bet mRNA in patients with rapid and slow disease progression but an increase of the IFN-γ mRNA only in patients with rapid progression of the disease. Very recently, a functional study focused on the effect of IL-17A on control or FUS-mutated MNs. The authors showed that IL-17A decreased MN survival and altered the neurite network in a dose-dependent manner and that the treatment with anti-IL17A and anti-IL-17A receptor helped to rescue dying MNs. All these observations were specific to IL-17A, as IL-17F treatment was not toxic to MN (Jin et al., 2021).

Few studies reported a decrease of Th2 cells or Th2-associated markers in blood of ALS patients (Henkel et al., 2013; Jin et al., 2020). In the same way as for Treg cells, an inverse correlation of GATA-3 and IL-4 mRNA expressions was found with disease rate progression (Henkel et al., 2013). On the contrary, Saresella et al. (2013) showed an increase of CD4 GATA3 cells in patient’s blood and no change in CD4 IL-4 cells, while another study described a positive correlation between the percentage IL-13 cells within CD4 T cells and the disease progression rate and an inverse correlation between the percentage IL-13 cells within the CD4 T cell population and the ALSFRS-R score (Shi et al., 2007). These results are of particular interest as the two major Th2 cytokines, IL-4 and IL-13, have been shown to increase CCL-2 production by activated primary rat microglia and by an activated human monocytic cell line THP-1 (Szczepanik et al., 2001). Whether Th2 cells or Th2 cytokines have a neuroprotective or a neuro-inflammatory role seems to depend on the context.

Taken together, these data show that the composition of the total T cell population is modified in ALS patients compared to controls. The balance between the different subsets of Th appears to evolve over disease progression. Interestingly, specific correlations seem to exist between expressions of specific mRNA Th subtypes and ALS patient’s disease progression rate (Henkel et al., 2013). These markers could be of great interest as new biomarkers to monitor disease progression rate in ALS patients.

In FTD patients, while one study described a decrease of total T cell percentages in blood samples (Busse et al., 2017), another study described a specific decrease of cytotoxic T lymphocyte antigen-4 (CTLA-4) CD4 T cells and no change of CD28 CD4 T cell frequency in patients’ blood (Santos et al., 2014). CD28 is a co-stimulatory immune checkpoint that promotes T cell activation, proliferation and response, whereas CTLA-4 is an immune checkpoint molecule exerting an inhibitory function on T cell proliferation and function. These two molecules compete for the same CD80/CD86 receptor (Chambers et al., 2001). Hence, this CTLA-4 deficiency could suggest a possible exacerbated activation of these CD4 T cells in FTD patients. Despite these studies, little is known about the role CD4 T cells in FTD. Given their rising importance in ALS, it would be worth investigating Th subsets in more details.

##### CD8 T Cells

The main function of CD8 T cells, also called cytotoxic T cells (CTL), consists in the elimination of cells infected with intracellular pathogens like viruses but also in elimination of tumor cells. CD8 T cells have been shown to play a determinant role in neurodegenerative diseases like multiple sclerosis (Huseby et al., 2012), but studies about their involvement in ALS remains sparse.

Similarly to Th cells, CD8 T cell infiltration were observed in the spinal cord and in the brain of ALS patients (Kawamata et al., 1992; Engelhardt et al., 1993; Fiala et al., 2010). Contradictory results exist on the percentages of CD8 T cells in ALS patient’s blood. Some studies showed an increase (Rentzos et al., 2012; Jin et al., 2020) while others suggested a decrease (Mantovani et al., 2009) or no change (Murdock et al., 2017). In SOD1*^G93A^* mice, CD8 T cells were shown to progressively infiltrate spinal cords (Chiu et al., 2008; Beers et al., 2011; Figueroa-Romero et al., 2019), while Beers et al. (2008) observed CD8 T cell infiltration only at disease end-stage in SOD1*^G93A^*/CD4^–/–^ mice. In 2018, a role of CD8 T cells and MHC-I in disease progression was described. In SOD1*^G93A^* mice defective for CD8 T cells and expressing little MHC-I, a dual role of MHC-I was shown with specific pathogenesis in the CNS and the PNS. In the CNS, without CD8 T cell infiltration and MHC-I microglia, inflammation was reduced, paralysis of forelimbs was delayed and survival of mice was extended. On the contrary in the PNS MN’s axons stability was affected leading to the acceleration of muscle atrophy (Nardo et al., 2018). This suggested that MHC-I-dependent interaction between either CD8 T cells or microglia is a key factor triggering neuroinflammation, and also that a slow-down of disease progression may be obtained through activation of the MHC-I signaling in the periphery to protect the axon-muscle connectivity.

In FTD, CD8 T cells were detected in the cortex of patients with tau*^P301L^* mutation (Laurent et al., 2017). Same observations were made in a mouse model of tauopathy and interestingly the depletion of peripheral T cells using an anti-CD3 antibody abolished CD8 infiltration in the cortex but also restored cognitive capacity of mutant mice, suggesting a crucial role of these T cells in cognitive impairment (Laurent et al., 2017).

#### B Lymphocytes

B cells are the center of adaptive humoral immunity and, by antigen-specific immunoglobulin production, they mediated extracellular pathogen elimination. Evidences of B cell implication in ALS is very limited. Auto-antibodies against proteins of spinal cord cells were detected in the serum and the CSF of some ALS patients (Niebroj-Dobosz et al., 2004, 2006; Puentes et al., 2014). Auto-antibodies have also been detected in SOD1*^G93A^* mice without any impact on disease progression (Naor et al., 2009). It remains unclear if auto-antibodies may be a consequence of MN death and if they could participate to MN degeneration and inflammation aggravation. A recent study investigated the impact of regulatory B (Breg) cell adoptive transfer on disease progression in SOD1*^G93A^* mice (Pennati et al., 2018). Similarly to Treg, Breg are immunosuppressive B cells involved in immune homeostasis and tolerance maintenance (Rosser and Mauri, 2015; Peng et al., 2018). In mutant mice, Breg adoptive transfer increased Treg cell percentage in the CNS at 5 months after the transfer, but no impact on the survival was reported (Pennati et al., 2018). It would be of particular interest to understand why and how Breg cells impact Treg cells. As Treg cells are decreasing overtime in ALS patients, being able to increase their numbers, even in an indirect manner, could be of interest to delay disease progression. In FTD patients, only one study reported a decrease of B cell percentage in the blood of patients (Busse et al., 2017). More investigations are now necessary to understand their role in FTD. Indeed, and more globally, current studies about immunity in FTD were more focused on dementia and FTD phenotypes were mainly compared to AD phenotypes. Considering the common spectrum of ALS and FTD, more comparative studies would help to understand whether these two neurodegenerative diseases share some immune deregulation and how inflammation really affects disease progression in FTD.

In conclusion, what is better understood today is which type of immune cell could be implicated in the disease, where it could play a role in the disease (Figure 1 and Table 3) and what they could secrete to protect/attack neurons or to eliminate debris and dead cells (Table 1). However, which cells are responsible for a specific secretion as well as which cells respond to specific factors remains largely elusive. Direct interactions between neurons and immune cells are still not well understood, as it remains impossible to have access to human brain and spinal cord tissues in alive patients.

**TABLE 3:** Publications studying immune cell involvement in ALS and FTD pathologies.

Type of immunity	Immune cell type	Model	ALS	FTD
Innate immunity	Macrophages	Animal model	− Slow down disease progression and increased survival when decreasing human mutant SOD1 expression in macrophage and microglia of mutant SOD1 mice (Beers et al., 2006; Boillée et al., 2006). − Decrease of sciatic nerve macrophage activation and CNS microglia activation when replacing SOD1*^G93A^* by more trophic macrophaged in the periphery in SOD1G93A mice (Chiot et al., 2020). − Delay of SOD1*^G93A^* mouse disease onset when replacing SOD1*^G93A^* macrophages by more trophic ones at the pre-symptomatic stage (Chiot et al., 2020). − Increase of SOD1*^G93A^* mouse survival when replacing SOD1*^G93A^* macrophages by more trophic ones at disease onset, but not at the pre-symptomatic stage (Chiot et al., 2020). − C9orf72 loss in mice leads to lysosomal trafficking defects, altered macrophages responses and increased inflammation (ORourke et al., 2016). − * Robust activation of CD169/CD68/Iba1+ macrophages in SOD1*^G93A^* and SOD1*^G37R^* transgenic mouse models, and infiltration along axons (Chiu et al., 2009).	− Hyper-activated macrophages after spinal cord injury in GRN^–/–^ mice (Naphade et al., 2010). − Hyper-activation of the innate immune system and increased expression of IL-6 and IL-1β in mice with C9orf72 deficiency (ORourke et al., 2016).
		Human patient	− Post-mortem studies: increased macrophages in the ventral root, demyelination, axonal degeneration, abnormal motor end plates, axonal sprouting, atrophic muscles (Bjornskov et al., 1984; Tandan and Bradley, 1985; Chiot et al., 2020). − * Possible infiltration in spinal cord tissues (Fiala et al., 2010).	− * Decreased HLA-DR expression by peripheral circulating CD14+ cells (Busse et al., 2017).
	Microglia	Animal model	− Slow down disease progression and increased survival when decreasing human mutant SOD1 expression in macrophage and microglia of mutant SOD1 mice (Beers et al., 2006; Boillée et al., 2006). − Decreased microglial reactivity when replacing peripheral SOD1*^G93A^* macrophages by more trophic macrophages (Chiot et al., 2020). − Neuroprotective effects of reactive microglia in a TDP-43 mouse model (Spiller et al., 2018). − C9orf72 loss in mice lead to lysosomal trafficking defects, altered microglial response and increased neuroinflammation (ORourke et al., 2016). − * Two-photon laser scanning microscopy: increased microglial reactivity in preclinical stages and switch to less motile ameboid microglia during clinical phases (Dibaj et al., 2011). − * Purinergic receptors involvement in SOD1*^G93A^* microglial cells hyper reactivity (Apolloni et al., 2013).	− GRN deficient mice display aberrant microglial activation, increased CD68^+^ cells, and TNF-α circulation (Moisse et al., 2009; Naphade et al., 2010; Tanaka et al., 2013; Lui et al., 2016). − C9orf72 deficiency leads to hyper activation of innate immune system, increased expression of IL-6, IL-1β, and upregulation of inflammatory genes in the spinal cord (Atanasio et al., 2016; ORourke et al., 2016; Sullivan et al., 2016; Moens et al., 2017; Beckers et al., 2021).
		Human patient	− Post mortem studies: increased microglial activation, presence of CD68+ macrophages in the vicinity of motor neurons (Brownell et al., 1970; Kawamata et al., 1992; McGeer et al., 1993; Nihei et al., 1993; Schiffer et al., 1996; Henkel et al., 2004; Sugiyama et al., 2013; Saberi et al., 2015). − PET scanning: increased TSPO binding only in motor cortices compared to controls (Zürcher et al., 2015; Alshikho et al., 2016, 2018; Ratai et al., 2018). − * Increased expression of ionotropic P2X7 in activated microglia in post mortem spinal cords, which serve as receptor for neuronal degeneration inducing inflammatory reaction (Yiangou et al., 2006). − * Increased expression of MCP1 in motor cortex microglia (Jara et al., 2017).	− Symmetrical convolutional atrophy in frontal and anterior lobes, gliosis (Ikeda, 2000; Tolnay and Probst, 2001). − PET scanning: TSPO binding in cortical frontal, mesial temporal, subcortical regions, prefrontal cortex, hippocampal, and parahippocampal regions (Cagnin et al., 2004; Turner et al., 2004; Miyoshi et al., 2010; Chew and Atassi, 2019).
	Monocytes	Animal model	− Increase of classical (CD14^+^, CD16^++^) and decrease of non-classical (CD14^++^, CD16^–^) monocytes in SOD1*^G93A^* mice (Butovsky et al., 2012; Zondler et al., 2016).	No information
		Human patient	− Increase of classical (CD14^+^, CD16^++^) and decrease of non-classical (CD14^++^, CD16^–^) monocytes in sALS, fALS patients but also in pre-symptomatic ALS mutant carriers (Butovsky et al., 2012; Zondler et al., 2016). − Differential profiles of gene expression in ALS monocytes (Zondler et al., 2016; Zhao et al., 2017)	− Decreased expression of CCL3 in CD14+ monocytes (Torres et al., 2012). − Decreased HLA-DR expression by peripheral circulating CD14+ cells (Busse et al., 2017).
			− * Upregulation of genes involved in leukocyte extravasation ITGB2, INPP5D, SELL, ICAM1 (Andrés-Benito et al., 2017). − * Decreased CCR2 expression on circulating monocytes in sALS patients (Zhang et al., 2006; Mantovani et al., 2009).	− Decreased expression of CCL3 in CD14+ monocytes (Torres et al., 2012). − Decreased HLA-DR expression by peripheral circulating CD14+ cells (Busse et al., 2017).
Main intermediate between innate and adaptive immunities	Dendritic cells	Human patient	− Observation of DC in cortico-spinal tracts of patients (Henkel et al., 2004). − Higher number of DC transcripts in the spinal cord of patient with a rapid disease progression compared to patient with a slower disease progression (Henkel et al., 2004). − Decrease numbers of DC in patient blood (Rusconi et al., 2017).	No information
Innate immunity	Mast cells	Animal model	− Accumulation of mast cells in the spinal cord of SOD1*^G93A^* mouse and rat models (Kovacs et al., 2021). − Recruitment along the degenerating nerve in SOD1*^G93A^* rat model (Trias et al., 2017).	No information
		Human patient	− Observation of mast cells in spinal cord of patients (Graves et al., 2004). − Observation of mast cells in the gray matter of spinal cord tissues, near to altered microvascular elements and surrounding motor neuron cell bodies (Kovacs et al., 2021).	
	NK cells	Animal model	− NK cells infiltration in SOD1*^G93A^* mouse CNS (Garofalo et al., 2020). − Decrease numbers of microglia and increase of Treg cells in spinal cord’s ventral horns of NK-deficient mice (Garofalo et al., 2020).	No information
		Human patient	− Observation of NK cells in spinal cord and motor cortex of patients with sporadic form of ALS (Garofalo et al., 2020). − Increase of NK cells in patients’ blood (Gustafson et al., 2017; Jin et al., 2020).	No change of NK cell percentage in patients’ blood (Busse et al., 2017).
Adaptive immunity	CD4 T cells	Animal model	− Increase numbers of Th1 and Th17 cells at late stage of the disease and Treg cells decrease during disease progression in SOD1*^G93A^* mice (Beers et al., 2011; Zhao et al., 2012).	No information
		Human patient	− Observation of CD4 T cells in spinal cords of patients (Kawamata et al., 1992; Engelhardt et al., 1993). − Lower FoxP3 (Treg) and Gata3 (Th2) mRNA levels in patients with rapid disease progression compared to patients with slow disease progression (Henkel et al., 2013). − Higher IFN-γ (Th1) level in patients with rapid disease progression compared to patients with slow disease progression (Henkel et al., 2013). − *Th1 cells*: Increased Th1 cell percentages in patients’ blood (Saresella et al., 2013; Jin et al., 2020). − *Th2 cells*: Mainly classified as neuroprotective but it seems to depend on the context (increase, decrease, or no change) (Shi et al., 2007; Henkel et al., 2013; Saresella et al., 2013; Jin et al., 2020). − *Th17 cells*: Increased Th17 cell percentages in patients’ blood (Rentzos et al., 2010; Saresella et al., 2013; Jin et al., 2020). Functionally, IL-17A decreased survival and altered the neurite network of control and FUS-mutated motor neurons (Jin et al., 2021). − *Treg cells*: Decreased Treg cell percentages in patients’ blood (Mantovani et al., 2009; Henkel et al., 2013; Saresella et al., 2013; Sheean et al., 2018; Jin et al., 2020) and functionally, lower suppressive capacity of ALS patients’ Treg compared to healthy control ones (Beers et al., 2017).	− Decrease of CD4 T cells specifically expressing CTLA-4 (inhibitory immune checkpoint) in patients’ blood (Santos et al., 2014).
	CD8 T cells	Animal model	− CD8 T cell infiltration in SOD1*^G93A^* mouse CNS (Beers et al., 2008, 2011; Chiu et al., 2008; Figueroa-Romero et al., 2019). − Decrease of inflammation and increase of spinal cord motor neurons’ survival in SOD1*^G93A^* CD8-deficient mice (Nardo et al., 2018).	− Abolishment of CD8 infiltration in spinal cord and restoration of cognitive capacity in a THY-Tau22 mouse model when T cells are depleted in periphery (Laurent et al., 2017).
		Human patient	− Observation of CD4 T cells in spinal cords and brains of patients (Kawamata et al., 1992; Engelhardt et al., 1993; Fiala et al., 2010). − No consensus about an increase or a decrease of the numbers of CD8 T cells in patients’ blood (Mantovani et al., 2009; Rentzos et al., 2012; Murdock et al., 2017; Jin et al., 2020).	− Observation of CD8 T cells in the cortex of FTD patients with tau*^P301L^* mutation (Laurent et al., 2017).
	B cells	Animal model	− Detection of autoantibodies in SOD1*^G93A^* mice (no impact on survival) (Naor et al., 2009). − No impact of B cell deficiency on disease progression (Naor et al., 2009). − Increase percentages of Treg cells at a specific timepoint after Breg cell adoptive transfert, however, no impact on mouse survival (Pennati et al., 2018).	No information
		Human patient	− Detection of autoantibodies against proteins of spinal cord’ cells in CSF of patients (Niebroj-Dobosz et al., 2006). − Detection of autoantibodies against gangliosides (Niebroj-Dobosz et al., 2006) and neurofilament proteins (Puentes et al., 2014) in patients’ blood.	− Decreased B cell percentages observed in patients’ blood (Busse et al., 2017).
			*: publication not included in the text	

*This table recapitulates what is known about dysfunction of immune cells in ALS and FTD, in both human and animal models. Mast cells, monocytes, phagocytes (macrophages and microglia), and NK cells belong to innate immunity. T cells (CD4 and CD8 T cells) and B cells belong to adaptive immunity. Dendritic cells make the link between innate and adaptive immunities. ALS, amyotrophic lateral sclerosis; C9orf72, chromosome 9 open reading frame 72; CCL3, C-C motif chemokine ligand 3; CCR2, C-C motif chemokine receptor 2; CD4, cluster of differentiation 4; CD8, cluster of differentiation 8; CD14, cluster of differentiation 14; CD16, cluster of differentiation 16; CD68, cluster of differentiation 68; CNS, central nervous system; CSF, cerebrospinal fluid; CTLA-4, cytotoxic T-lymphocyte associated protein 4; fALS, familial amyotrophic lateral sclerosis; FoxP3, forkhead box P3; FTD, frontotemporal dementia; FTLD, frontotemporal lobar degeneration; Gata-3, GATA binding protein 3; GRN, granulin precursor; ICAM1, intercellular adhesion molecule 1; IFN-γ, interferon-gamma; INPP5D, inositol polyphosphate-5-phosphatase D; ITGB2, integrin subunit beta 2; PNS, peripheral nervous system; sALS, sporadic amyotrophic lateral sclerosis; SELL, selectin L; SOD1, superoxide dismutase 1; TDP-43, transactive response DNA binding protein 43; Th, T helper cell; Treg, regulatory T cell.*

## Clinical Trials to Modulate the Immune System

The complexity of the whole immune system and the numerous cell types involved in the different inflammatory pathways make difficult the understanding of the real impacts of therapeutic drugs on disease phenotypes. However, with the urgent need to find a cure or even a treatment to delay disease progression, clinical trials were launched and since the late 1990s several immune modulatory drugs or cell therapies have been tested in clinical trials for ALS.

### Immune Modulatory Drugs

**FIGURE 2:**
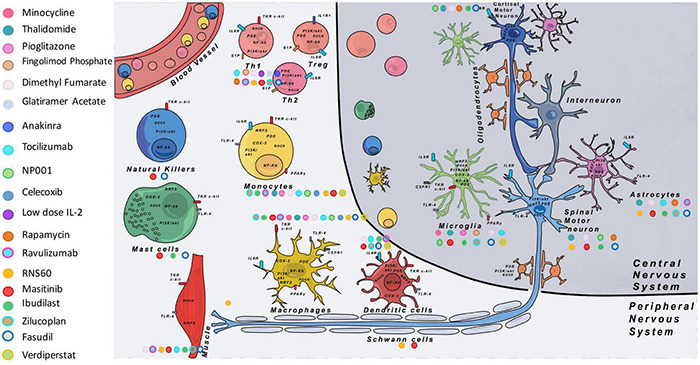
Main immune modulatory molecules tested in ALS clinical trials. On the left are listed the different tested molecules with their corresponding colors. On the figure each color dot next to the different cell types shows on which cell the immune modulatory drug is supposed to have an effect. Some cell types targeted by the drugs were well studied in the literature (see Table 4), but in some cases we completed data with theoretical targets (depending on the expression of the target on specific cell types indexed with proteinatlas.org, and for which no information in the literature was found). COX-2, cyclooxygenase-2; CSF1R, colony stimulating factor 1 receptor; IL-1R1, interleukin 1 receptor 1; IL-2, interleukin-2; IL-6R, interleukin 6 receptor; NF-κB, nuclear factor kappa-light-chain-enhancer of activated B cells; NRF2, nuclear factor erythroïd-2-related factor 2; PDE, phosphodiesterase; PGE2, prostaglandin E2; PI3K, phosphoinositide 3-kinase; PPARγ, peroxisome proliferator-activated receptor gamma; ROCK, Rho-associated protein kinase; S1P, sphingosine-1-phosphate receptor; TKR, tyrosine-kinase receptor; TLR-4, Toll-like receptor 4.

As shown in Table 4 and Figure 2, a large majority of considered immune modulatory drugs in ALS clinical trials are repositioned molecules previously tested in other diseases harboring inflammation. Their mechanisms of action are not presented here as they were described previously in details (Khalid et al., 2017). Table 4 lists trials according to their progress. Among tested molecules, most are antagonists of pro-inflammatory pathways reported to be deregulated in ALS patients. While some tested molecules induced adverse effects, most of them demonstrated safety and tolerability but their efficacy remains limited or negative. To date, most of the proposed molecules have a very large panel of targets and act on signaling pathways shared by many cells. While the two molecules accepted on the market (Riluzole and Edaravone) claim to act on neuroprotective functions for MNs, it turns out that for immune modulating trials the targets are broad with possible uncontrolled impacts on the disease pathology (Table 4). Today, eleven trials are either recruiting and/or active, targeting one or several molecules and pathways. Among these trials, one ongoing phase II aims to target T cells with low IL-2 dose (ld-IL-2). This trial has already shown some encouraging results with increased Treg cells (Camu et al., 2020). In another phase II trial, RNS60, an anti-inflammatory drug, targets the p-Akt pro-survival pathway and NF-κB (Paganoni et al., 2019). Compared to the ld-IL-2 trial the latter has 6 defined cell targets and 3 possible others, a non-specificity that could be responsible for uncontrolled modulations of several pathways. Fortunately, adverse effects were not reported. A Phase II/III trial (Ibudilast) uses a small molecule already tested in patients with multiple sclerosis. Contradictory results were reported and further studies are now necessary to understand the exact impact of Ibudilast on patients (Chen et al., 2020; Babu et al., 2021). Finally, a phase III trial (Ravulizumab) uses a monoclonal antibody targeting the C5 complement to decrease inflammation. Disappointing results were announced in August 2021 and the trial was discontinued because of a lack of efficacy.

**TABLE 4:** List of the different immune modulatory therapeutic approaches clinical trials.

Molecule name	Type of molecule	Target	Effects	Theoretical targeted cell types	Clinical phases	Clinical trials conclusions	Used for other diseases	References
Minocycline (Minocin)	Small molecule	Apaf-1? Apoptotic machinery	↘ caspase-1, caspase-3, iNOS, MAPK	• **MG**	Phase III (completed 2007)	Negative effects on patients.	Acne vulgaris Infections Asthma	Chen et al., 2000; Wang et al., 2003; Gordon et al., 2007
				• MP				
Thalidomide (Contergan/Thalomid)	Small molecule	3′-UTR of TNF-α mRNA	↘ IL-1β, IL-6, IL-10, IL-12, TNF-α	• **MG**	Phase II (completed 2007)	No positive effects and can cause adverse effects.	Multiple myeloma	Clinical trial: NCT00140452 Stommel et al., 2009; Jung et al., 2019
				• Monocytes				
				• MP				
				• Other CNS cells (endothelial cells, neuronal cells, astrocytes)				
Pioglitazone (Actos)	Small molecule	(+) PPAR?	↘ Inflammatory mediators modulate transcription of insulin responsive genes stimulates adipocytes differentiation	• **MP**	Phase II (completed 2015)	The interim analysis showed no tendency in favor of the verum group. Therefore it was decided to stop the study prematurely.	Type 2 diabetes	Clinical trial: NCT00690118 Dupuis et al., 2012; Jawaid et al., 2014
				• **Monocytes**				
				• **Adipocytes**				
				• MG				
Fingolimod (Gilenya)	Small molecule	(+) S1P	Decrease S1P1 Prevents lymphocytes egress from lymphoid tissues	• **CD4 lymphocytes and Treg**	Phase Iia (completed 2015)	Safe and well tolerated.	Multiple sclerosis Chronic inflammatory Demyelinating polyneuropathy	Potenza et al., 2016; Berry et al., 2017
				• **MG**				
Dimethyl Fumarate	Small molecule	(+) NRF2 (−) GAPDH	Antioxidant response ↘ Th1 ↖ Th2 ↖ Type II DC ↖ Aerobic glycolysis	• **T cells (including Treg)**	Phase II (completed 2019)	Assessing efficacy and safety.	Psoriasis Multiple sclerosis	Clinical trial: ACTRN12618000534280 Gross et al., 2016; Schlöder et al., 2017; Kornberg et al., 2018; Piroli et al., 2019; Holm Hansen et al., 2020; Vucic et al., 2020
				• DC				
				• MP				
				• Monocytes				
				• MG				
				• Other CNS cells (neurons, astrocytes)				
				• Muscles				
Glatiramer Acetate (Copaxone)	Small molecule	Mimicks MBP	Modulate T cells reactivity	• **T cells (including Treg)**	Phase II (completed 2008)	Safe and tolerable. Glatiramer acetate at dose of 40 mg/day did not show beneficial effect in ALS.	Multiple sclerosis	Clinical trial: NCT00326625 Meininger et al., 2009
Tocilizumab (Actemra)	Monoclonal antibody	(−) IL-6R	↘ IL-6	• **MP**	Phase III (completed 2018)	Safe and tolerable, reduces c reactive protein concentration in plasma and CSF of ALS patients.	Rheumatoid arthritis Systemic juvenile idiopathic arthritis Castleman’s disease Giant cell arteritis Cytokine release syndrome	Clinical trial: NCT02469896 Mizwicki et al., 2012; Fiala et al., 2013; Sheppard et al., 2017; Milligan et al., 2021
				• **B cells**				
				• **T cells**				
				• **Osteoclasts**				
				• **Monocytes**				
				• DC				
				• MG				
				• Other CNS cells (astrocytes, neuronal cells)				
				• Muscle				
Celecoxib (Celebrex)/ Ciprofloxacin	Small molecule	(–) Cox-2 bacterial DNA gyrase and topoisomerase IV.	?↘ PGE2	• **Monocytes**	Phase I (combined therapy with Ciprofloxacin; active, not recruiting)	800 mg/day was safe and tolerable but did not show beneficial effects for ALS patients. New combined trial with Ciprofloxacin is ongoing.	**Celecoxib** Osteoarthritis Rheumatoid arthritis Ankylosing spondylitis Acute pain Musculoskeletal pain Familial adenomatous polyposis **Ciprofloxacin** Infections	Clinical trial: NCT04090684 Cudkowicz et al., 2006; Gong et al., 2012; Na et al., 2013; Nagano et al., 2017
				• **MP**				
				• **Osteoblasts**				
				• Smooth muscles				
				• Granulocytes				
				• Bone marrow				
				• MG				
				• Other CNS cells				
NP001	pH-adjusted IV formulation of purified sodium chlorite	Chlorite is converted into taurine chloramine ↘ NF-kB expression	Modulation of monocytes activation and ↘ IL-1β	• **MP**	Phase IIb (completed)	Safe but do not significantly slow progression of the disease. Study lacks precision to exclude important effect.	/	Miller et al., 2014, 2015, 2018
				• **Monocytes**				
				• MG				
				• Other CNS cells				
Ravulizumab (Ultomiris)	Monoclonal antibody	(−) Complement 5	↘ Inflammation	• **MP**	Phase III (stopped in 2021)	Pivotal study. Role of complement-mediated damage in ALS patient is evident, lack of efficacy.	Paroxysmal nocturnal hemoglobinuria Atypical hemolytic uremic syndrome	Clinical trial: NCT04248465 Lee et al., 2017; Wang et al., 2017
				• **Monocytes**				
				• **Granulocytes**				
				• **T cells**				
				• MG				
				• DC				
				• B cells				
				• Astrocytes				
				• Muscle				
Anakinra (Kineret)	Monoclonal antibody	(−) IL-1R	↘ IL-1α, IL-1β	• **T cells**	Phase II (recruiting since 2011)	Safe, tolerable. No reduction on disease progression, increased serum inflammatory markers.	Rheumatoid arthritis Cryopyrin-associated periodic syndrome Macrophage activation syndrome Schnitzler’s syndrome Hemophagocytic lymphohistiocytosis	Clinical trial: NCT01277315 Cohen, 2004; Maier et al., 2015
Low dose-IL-2 (Aldesleukin)	Small molecule	(−) IL-2R, IL-15Rβ	Promotes Treg differentiation + pleiotrope actions on the immune system	• **T cells**	Phase II (active, not recruiting)	Safe, increased Tregs, no differences on plasma NFL.	Malignant melanoma Renal cell cancer Autoimmune disease	Clinical trial: NCT03039673 Camu et al., 2020
				• Cells in the CNS				
Rapamycin (Rapamune)	Small molecule	(−) mTOR	Promotes Treg differentiation ↖ Autophagy	• **T cells**	Phase II (active, not recruiting)	Already well known pharmacokinetics, safety and tolerability.	Organ transplant rejection Lymphangioleiomyomatosis	Clinical trial: NCT03359538 Staats et al., 2013; Madill et al., 2017; Mandrioli et al., 2018
				• **Neuronal cells**				
				• **Astrocytes**				
RNS60	Experimental nanostructured drug containing various oxygen nanobubbles	(+) p-Akt pro survival pathway (−) NF-κB	↘ Mitochondrial alteration and oxidative stress ↖ Anti-inflammatory phenotype ↖ IL-4	• **MN**	Phase II (active, not recruiting)	Long-term administration was safe and well-tolerated.	/	Clinical trial: NCT03456882 Vallarola et al., 2018; Paganoni et al., 2019
				• **NMJ**				
				• **T cells**				
				• **Muscle**				
				• **MP**				
				• **MG**				
				• Astrocytes				
				• Schwann cells				
				• Monocytes				
Masitinib (Masivet)	Small molecule	(−) TKR c-kit, CSF1R, tyrosine protein kinase Lyn, proto-oncogene tyrosine protein kinase Fyn	↘ Proliferation of MG and MP Other effects unknown?	• **Mast cells**	Phase III (active, recruiting)	Safe and tolerable. Slows ALSFRS-R decline of 27% after 48 we of treatment, VFC of 22% and ALSAQ-40 of 29%.	European approval of Masitinib for treatment of ALS (Alsitek) was refused in 2018	Trias et al., 2016, 2017, 2018; Mora et al., 2020
				• **MP**				
				• **MG**				
				• **Neutrophils**				
				• Schwann cells				
				• NK				
				• Muscle				
				• Monocytes				
				• DC				
				• T cells				
				• Other CNS cells (high level of the target in the cerebellum, astrocytes, neuronal cells)				
Ibudilast	Small molecule	(−) PDE 3, 4, 10, 11 (−) TLR-4, NO (−) MIF	↘ IL-1β, TNF-α, IL-6, ROS ↖ IL-10, NTF (BDNF, NGF, NT-4)	• **MP**	Phase II/III (active, recruiting)	Safe and tolerable. Sentinel ALS muscle strength was significantly reduced 2 we post cessation of the treatment.	Asthma Allergic conjunctivitis Hay fever Stroke	Gibson et al., 2006; Cho et al., 2010; Cox et al., 2013; Brooks et al., 2015, 2016, 2017; Ha et al., 2019; Oskarsson et al., 2020; Babu et al., 2021
				• **Other CNS cells (astrocytes, neuronal cells)**				
				• **MG**		Another study conclude that 100 mg/day in ALS, no significant reductions in motor cortical glial activation over 12–24 we, CNS neuroaxonal loss (NFL measure) over 36–40 we. Future pharmacokinetic and dose finding studies required.		
				• Monocytes				
				• Neutrophils				
				• DC				
				• Granulocytes				
				• Mast cells				
				• Muscle				
				• Bone marrow				
Zilucoplan	Small molecule	(−) Complement 5	↘ Inflammation	• **MP**	Phase II (active, recruiting)	Implication of complement in ALS patients.	/	Clinical trial: NCT04297683 Lee et al., 2017; Wang et al., 2017
				• **Monocytes**				
				• **Granulocytes**				
				• **T cells**				
				• MG				
				• DC				
				• B cells				
				• Astrocytes				
				• Muscle				
Fasudil	Small molecule	(−) Rho kinase	↘ Pro-inflammatory cytokines ↖ Axonal regeneration, NMJ, actin cytoskeleton plasticity ↖ Akt pro-survival pathway	• **MG**	Phase IIa (active, recruiting)	Previous reports in the first compassionate use of Fasudil show in *n* = 3 patients slow VFC significant increase in one of the patients and safety and tolerability.	Stroke Subarachnoid hemorrhage Pulmonary hypertension	Clinical trial: NCT03792490 Tönges et al., 2014; Lingor et al., 2019; Koch et al., 2020
				• **Muscle**				
				• Other CNS cells (neuronal cells, endothelial cells, astrocytes)				
				• MP				
				• DC				
				• NK				
				• T cells				
				• Mast cells				
Verdiperstat	Small molecule	(−) MPO enzyme	↘ Oxidative stress, inflammation	• **Monocytes**	Phase III (enrolling by invitation)	/	/	Clinical trial: NCT04436510 Patnaik et al., 2020
				• **DC**				
				• **Neutrophils**				
				• **MG**				
				• **MP**				
				• Bone marrow				

*Main targets are listed for each molecule. This may be non-exhaustive as some molecules have very large spectrum of action still not well described. The main effects are presented similarly. The listing is based on actual literature and may evolve with time. Targeted cell types are presented. In blue bold are the cell types predicted to be targeted by the molecule in accordance with the literature. In black are other cell types expressing the target and that may also be impacted by the treatment. The expression of each cellular target was investigated via proteinatlas.org in order to index cell types susceptible to be targeted by the drug. Main focus was driven on immune related cell types. Trials are sorted depending on their clinical trial ongoing status. 3′ UTR, 3′ untranslated transcribed region; ALS, amyotrophic lateral sclerosis; ALSAQ-40, Amyotrophic Lateral Sclerosis Assessment Questionnaire; ALSFRS-R, Amyotrophic Lateral Sclerosis Functional Rating Scale; Apaf-1, apoptotic protease activating factor 1; BDNF, brain-derived neurotrophic factor; C5, complement 5; CNS, central nervous system; CSF, central nervous system; COX-2, cyclooxygenase-2; CSF1R, colony stimulating factor 1 receptor; DC, dendritic cell; GAPDH, glyceraldehyde-3-phosphate dehydrogenase; IL-1α, interleukin-1 alpha; IL-1β, interleukin-1 beta; IL-1R, interleukin-1 receptor; IL-2R, interleukin-2 receptor; IL-4, interleukin-4; IL-6, interleukin-6; IL-6R, interleukin-6 receptor; IL-10, interleukin-10; IL-12, interleukin-12; IL-15R, interleukin-15 receptor beta; iNOS, inducible nitric oxide synthase; MAPK, mitogen-activated protein kinase; MBP, myelin basic protein; MG, microglia; MIF, macrophages migration inhibitory factor; MP, macrophage; MPO, myeloperoxidase; mTOR, mechanistic target of rapamycin; NF-κB, nuclear factor kappa-light-chain-enhancer of activated B cells; NFL, neurofilament light chain; NGF, nerve growth factor; NK, natural killer; NMJ, neuro-muscular junction; NO, nitric oxide; NRF2, nuclear factor erythroïd-2-related factor 2; NT-4, neurotrophin 4; NTF, neurotrophic factors; PDE, phosphodiesterase; PGE2, prostaglandin E2; PPARγ, peroxisome proliferator-activated receptor gamma; ROS, reactive oxygen species; S1P, sphingosine-1-phosphate receptor; S1P1, sphingosine-1-phosphate receptor 1; TLR-4, Toll-like receptor 4; TNFa, tumor necrosis alpha; TKR, tyrosine-kinase receptor; VFC, vital force capacity.*

There are several reasons that may explain the failure of each trial and they have to be taken into account in future trials. First, most of the clinical trials include patients with heterogeneous disease states [in terms of disease form (sporadic and familial), durations, and site of onset]. These are among reasons why most studies do not include ALS-FTD patients. Mechanisms involved in the pathological progression at different disease steps may vary between patients. Thus modulating inflammation with such heterogeneity may erase effects attributable to one subgroup. Edaravone is a good example. No beneficial effect was found from the initial clinical trial conducted in a heterogeneous ALS population. However, *post hoc* analysis demonstrated beneficial effects in a clinical subset of ALS patients (Okada et al., 2018; Abraham et al., 2019). Second, the inflammatory reaction in patients is suspected to switch from protective to deleterious during disease progression, suggesting that might exist a therapeutic window to ensure treatment efficacy in patients. This parameter remains very difficult to monitor as there are no validated biomarker able to assess the disease state. Fortunately, recent advances in imaging techniques bring new hope for future trials. For instance, a recruiting phase II clinical trial will evaluate the brain microglia response in ALS patients by TSPO binding following multiple doses of BLZ945, an antagonist of the CSF1 receptor, using PET with [11C]-PBR28 (NCT04066244). Other markers closely monitored and already measured in patients are concentrations of neurofilaments light chain (NF-L) and heavy phosphorylated chains (pNF-H) in blood and CSF (Gaiani et al., 2017; Benatar et al., 2018; Huang et al., 2020). A consensus on pNF-H as an early biomarker should give the possibility to better follow the effects of any drug on specific markers (Benatar et al., 2020). The future relies on the urgent need to identify specific biomarkers linked to different ALS forms and to be able to identify when a molecule or another can have a positive impact on the disease progression in each patient. Finally, as 90% of ALS cases are sporadic, the diagnosis is done when symptoms are already present and when inflammatory events are already well advanced. In this situation, having the possibility to follow markers in patients before any symptom appearance could bring very important insights. Chipika et al. (2020) have performed a systematic review of existing presymptomatic studies. They showed that studies suffered from small sample size, lack of controls, and rarely cohorts were followed until symptom manifestations. Objectives of these kind of studies are to be able to propose earlier diagnosis and thereby earlier therapeutic interventions or enrollment in clinical trials with homogenous sub-groups of patients. Personalized therapies are currently emerging, as exemplified by the antisens oligonucleotide Tofersen for SOD1 ALS patients (Miller et al., 2020) and even for presymptomatic SOD1 carriers in a new ongoing trial (NCT04856982^1^).

### Cell Transplantation

This second therapeutic approach implies transplantation of specific cell types in patients. More than 30 trials are listed on https://clinicaltrials.gov. Morata-Tarifa et al. (2021) provide in 2021 a meta-analysis of stem cell therapy in ALS. Among cell types available for transplantation, mesenchymal stem cells (MSC) have been the most widely tested autologous transplants through different techniques and route of administration (i.e., spinal cord, frontal lobe, and intrathecal) (Mazzini et al., 2008; Deda et al., 2009; Kim et al., 2014). MSC represent interesting cell candidates as they are easy to culture *in vitro* and are able to be differentiated into bone stroma, cartilage, ligament, and fat depending on the surrounding molecules (Deans and Moseley, 2000; Verboket et al., 2018). Moreover, these cells express cytokines and growth factors that could brought in therapeutic contributions for neuronal protection and act on local inflammation (Djouad et al., 2007; Suzuki et al., 2008). Besides MSC, bone marrow cells (BMC) were also transplanted in patients (Deda et al., 2009; Ruiz-López and Blanquer, 2016). These cells are a mixture of several cell types, containing lymphocytes, monocytes, and progenitor cells, giving them a strong regenerative potential (Verboket et al., 2018). Preclinical studies in animal models have shown safety and tolerability after transplantation of both cell types, as well as beneficial effects on pathogenic signatures (Kim et al., 2010; Lewis and Suzuki, 2014). In ALS trials, conclusions of the therapies have been difficult to draw, especially because of the small number of patients included (Mazzini et al., 2008; Deda et al., 2009; Karussis et al., 2010). Analysis of Morata-Tarifa et al. (2021) showed that the best results were obtained with intrathecal injections of MSC in patients but with only transient positive effects on clinical progression (ALSFRS-scores). This being, a major drawback of these transplantations has been that after all interventions respiratory functions were negatively impacted.

As described in the previous section, a clinical trial administrating IL-2 was launched in order to force the production of Tregs in ALS patients. No adverse effects were reported and an increased Tregs number was observed (Camu et al., 2020). Thus, transposition to cell therapy has been considered. Thanks to protocols allowing Tregs *ex vivo* expansion (Alsuliman et al., 2016), a phase I clinical trial was launched to test further transplantation of *ex vivo* expanded Tregs. First results suggested safety with no clear conclusion as the study was under-powered for efficacy and did not have placebo or controls. However, from the results observed in the 3 transplanted patients, Tregs transplantation induced an increase in Tregs percentages, amelioration of Treg suppressive functions and a slowing down in the decrease of the ALSFRS-score. Unfortunately, these effects disappeared between rounds of Treg cell injections (Thonhoff et al., 2018). Nonetheless, a phase II is currently undergoing including 12 patients with placebo controls, despite cumbersome protocols for patients.

The use of such autologous cell transplantation therapies could be very interesting as they appear more adapted to a large number of ALS patients. However, for all cell types proposed, it seems imperative today to come back to preclinical studies to optimize cell type choices and administration routes and to better understand cell distributions and their mechanisms of action.

## iPSC to Model Immune Reactivity in Amyotrophic Lateral Sclerosis and Frontotemporal Dementia

As described above, immune reactions implying several cell types arise during disease progression in patients with ALS and FTD. As inflammatory responses can be beneficial or deleterious depending on signals in their environment, performing longitudinal studies would be very informative. Moreover, deciphering specific mechanisms involved in direct or indirect interactions between affected neurons and the different immune cells, that could be new targets of clinical trials, have to be done. However, whereas blood sampling may allow access to some immune cells of patients, biopsies of the brain and the spinal cord regions in which neurons are affected and in which tissue resident or infiltrated cells have to be analyzed, are not possible. Thanks to the iPSC technology described 14 years ago, researchers can now have access to human pluripotent cells that can be differentiated in theory into any cell type of the body. “In theory” as we know now that each protocol to differentiate iPSC into specific cell subtypes necessitates years of technical developments. Nevertheless, iPSC offer the unique opportunity to study intrinsic defects in iPS-derived mutant cells and interactions between different cell types in 2D and 3D co-cultures.

### iPS-Derived Neurons

Until now, the vast majority of studies focused on iPS-derived neurons. Many reviews already present the unique capabilities of iPS-derived neurons to capture some key features of ALS and FTD in MNs and cortical neurons, respectively (Bohl et al., 2016; Lee and Huang, 2017; Hawrot et al., 2020; Lines et al., 2020). At first, protocols allowed only production of generic neurons with very poor purity. As technology advanced, many neuronal subtypes were able to be generated allowing to better understand the dichotomy between the ubiquitous expression of mutant genes in patients’ cells and the death of specific subtypes of neurons (Sances et al., 2016). Nevertheless, there are still improvements in progress to obtain in culture the most specific neuron subtypes. For example, for MNs, it is today possible to characterize in cultures if generated MNs are those of the lateral or medial motor column (LMC or MMC) (Amoroso et al., 2013). Recently Mouilleau et al. (2021) deciphered mechanisms allowing the expression of HOX genes involved in the identities of MNs along the rostro-caudal axis of the spinal cord. These different findings open the way to generate specific cervical to lumbar MNs of the LMC or MMC that may be differentially affected in ALS patients. Interactions between neurons and astrocytes were also reported in some publications (Lee and Huang, 2017; Lines et al., 2020) and different results were reported regarding toxicity of astrocytes toward MNs depending on the studied ALS form, showing the necessity to pursue investigations.

### Innate Immune Cells

#### Macrophages and Microglial Cells

Thanks to concomitant advances in the knowledge of macrophage biology, origin and diversity, protocols to generate macrophages have been progressively improved. For ALS, two subtypes of macrophages are of interest: microglial cells, the macrophages of the CNS, and peripheral macrophages located along the MN axon from the ventral root to the NMJ. For FTD, only microglial cells are of interest at first sight. To obtain macrophages/microglia from patients, first protocols were based on human monocytes isolated from blood and activated with cytokines *in vitro*. However, it has been shown that macrophages/microglia gradually loose their tissue identity in culture. It is now known that these macrophage-like cells are not capable to fully model tissue-resident macrophage populations that arise from embryonic precursors (Lavin et al., 2014; Hagemeyer et al., 2016; Lee et al., 2018). Moreover, macrophage activation exists as a spectrum of phenotypes and functional states challenging to reproduce in culture (Ginhoux et al., 2016).

As an alternative option to blood cells and as an infinite source of patient-specific cells, embryonic stem cells (ESC) and iPSC were tested for their capacity to be differentiated into macrophage-like cells. A great difficulty for such iPSC protocol developments was the capacity to generate tissue-resident macrophages and in particular macrophages of the CNS. Indeed, microglia and peripheral macrophages have different developmental origins and are in very different environments *in vivo*, suggesting that different protocols must be set up to mimic the diverse macrophage subtypes. Many reports and reviews describe and compare protocols allowing the generation of functional macrophage-like cells (Haenseler and Rajendran, 2019; Hasselmann and Blurton-Jones, 2020; Hedegaard et al., 2020; Lyadova et al., 2021). Clearly, iPSC require specific environmental signals for proper differentiation and maintenance of their identity (Bohlen et al., 2017; Gosselin et al., 2017), suggesting that for ALS and FTD studies, iPS-derived microglia have to be co-cultured with either MNs or cortical neurons, respectively.

iPS-derived macrophage/microglia monoculture phenotypic defects were observed in different models (Haenseler and Rajendran, 2019). Zhao et al. (2020) studied iPS-derived macrophages from C9ORF72^(G4C2)n^ patients and control subjects and compared their immune-suppressive functions. Their results demonstrated that immunosuppressive functions of ALS macrophages were similar to controls, suggesting that an ALS mutation had no influence on this function in macrophages. Some other reports described (i) 2D co-cultures of microglia with neurons to mimic their neural environment, (ii) microglia generation in brain or spinal cord organoids, or (iii) xenotransplantation of microglial-like cells (Hasselmann and Blurton-Jones, 2020; Fattorelli et al., 2021; Tan et al., 2021). Current reports in 2D cultures between neurons and iPS-derived microglia studied their morphology and migration, their inflammatory responses and clearance capacity. Similarly, in 3D cultures and in organoids, observations were about migration, activation, proliferation, and physical interactions of microglia with other cells (Haenseler and Rajendran, 2019). Most of current studies focused on microglia addition to organoids and modeling of Alzheimer’s or Parkinson‘s defects, making the proof that the different iPSC based models could bring new knowledge for ALS and FTD. Recently, the generation of microglia-like cells in human sensorimotor organoids derived from ALS iPSC was reported (Pereira et al., 2021), proving that these cells can arise and be phenotypically studied in these models.

#### Dendritic Cells, Mast Cells and Natural Killer Cells

To study DCs, it is possible, like for macrophages, to generate primary DC from peripheral blood-derived monocytes, and then to mature them into distinct DC subsets *in vitro* (Romani et al., 1994; Sallusto and Lanzavecchia, 1994). In their review of 2020, Ackermann et al. (2020) described the different protocols to generate DC and their subpopulations from iPSC. DCs were shown to be morphologically and functionally similar to their *in vivo* counterparts. Interestingly, the generation of DC offers the possibility to investigate their interactions with T cells derived from the same patient, as iPS-derived DC were shown to be able to stimulate allogeneic naïve T cells and autologous antigen-specific CD8+ T cells. These new cellular tools will be very helpful considering the limited availability of DC in blood and tissues.

Few protocols exist to differentiate iPSC into mast cells (Kovarova et al., 2010; Igarashi et al., 2018; Kauts et al., 2018; Ikuno et al., 2019). First protocols were long, with low yield, and a production of immature cells. Thanks to developmental studies (Gentek et al., 2018; Li et al., 2018), the most recent protocol uses a sequential co-culture system which allow short-term cultures and efficient production of scalable quantities of functional mature mast cells, exhibiting the strongest innate immune responses (Bian et al., 2021). Like macrophages, mast cells maturate in tissues under the influence of the local environment, suggesting that co-cultures with neurons might be the more relevant study model for ALS and FTD.

For NK cells, a recent review summarizes protocols to obtain these cells from human iPSC (Shankar et al., 2020). iPS-derived NK cells were shown to be phenotypically similar to primary NK cells as well as regarding their effector function. CD56 is a marker often used to define human NK cells (murine NK cells are CD56 negative). Two subpopulations (dim and bright) have been identified and play different roles (CD56 bright cells exhibit high cytokine secretion while CD56 dim exhibit high cytotoxicity). As it is unclear if these both cell types are generated from iPSC, further investigation will be necessary, before using those cells to conduct disease modeling.

### Adaptive Immune Cells

Whereas protocols to generate cells of the innate immune system were set up quite rapidly, the differentiation of iPSC into cells of the adaptive immune system appeared much more challenging (Montel-Hagen and Crooks, 2019). For B lymphocytes, first protocols just emerge and allow only to model the earliest stages of B lineage development (French et al., 2015; Böiers et al., 2018; Richardson et al., 2021). For T lymphocytes, whereas the first stages of iPSC differentiation into T progenitors were rapidly set up, some cell specificities are difficult to reproduce to obtain *in vitro* mature T lymphocytes. The particularity of the T-cell lineage is that the first stages of their differentiation occurs in the thymus. To reproduce the thymus environment and differentiate iPSC into T progenitors, co-cultures were performed between iPSC and stromal cells that express a Notch ligand. Notch was shown to be essential to induce T cell differentiation of progenitors, at the expense of B-cell differentiation. Recently, a stromal-cell free protocol was developed to generate the T cell lineage from blood-derived CD34-positive cells (Nianias and Themeli, 2019; Netsrithong et al., 2020), a strategy that has now to be transposed in iPSC differentiation protocols. Additionally, lymphocytes express at their surface different receptors forming a repertoire (TCRs for T cell receptors). The latter is crucial for the response of T lymphocytes in an antigen-specific manner to an unlimited number of unknown pathogens encountered throughout life. Interestingly iPS-derived T progenitors were shown to express a broad TCR repertoire (Chang et al., 2014), suggesting that each iPSC clone has its own TCR repertoire. Current limitations of the iPSC protocols rely in the maturation of T lymphocytes and their positive selection. This selection consists in the maturation of double positive CD4–CD8 lymphocytes into simple positive cells. Whereas iPSC protocols not clearly demonstrated their ability to mimic this positive selection, a recent improvement was proposed with the generation of 3D artificial thymic organoids which allowed the generation and maturation of conventional T cells (Montel-Hagen et al., 2019). Having in mind the different roles played by T cell subtypes in neurodegenerative diseases, it will be crucial now to generate IPS-derived Th1, Th2, Th17, or Treg cells. Once generated, those cells will be of great interest for ALS and FDT modeling.

### Modeling Immune Reactivity With Induced Pluripotent Stem Cell

In ALS and FTD, the original mono-centered pattern was for the neuron to degenerate and send out pain signals that alert cells in the environment. Thus for a long time the majority of studies focused on neurons analysis and how to avoid their degeneration (in patients, in animal models, and in cellular models) without too much consideration for other cells. In this context, iPSC-derived neurons made it possible to study different forms of ALS and FTD, including sporadic cases, and to show the progressive appearance of intrinsic defects in patients’ neurons. This is a major advantage of the iPSC technology that has made it possible to show different chronologies of appearance of neuronal dysfunctions from one patient to another (Fujimori et al., 2018) and that cannot be followed individually in patients. Thanks to iPSC modeling approaches, new therapeutic targets were identified and are currently being tested in patients (Wainger et al., 2014).

At the same time, genetic studies have shown that the majority of genes mutated in the different forms of ALS and FTD are expressed ubiquitously suggesting that all cells in the body can be altered in the pathology. The diagram has therefore evolved into an increasingly off-center diagram in which the neuron is no longer the central element but one element among others. Understanding the interactions between cells has therefore become a crucial issue in the understanding of neurodegenerative diseases in order to find new therapeutic avenues. Among the cells of interest are immune cells known to be activated in ALS and FTD. Unfortunately, as for neurons, these are cells that are difficult to access in patients. Thanks to protocols derived from iPSCs, it is now possible to study not only the intrinsic defects of these cells but also their interactions with neurons. It becomes possible to study inflammatory responses according to the normal or degenerative states of neurons, with the perspective of understanding how inflammation evolves and could therefore be controlled. These analyses, combined with those made in longitudinal studies in patients, could help identify the therapeutic window in which to intervene in the most beneficial way for patients.

## Conclusion and Perspectives

The main conclusion from decades of studies on patients and animal models is that ALS and FTD are heterogeneous diseases at several levels and have to be seen as a group of different diseases rather than a single one, at least regarding mechanisms leading to neuron degeneration. To date ALS or FTD patients are often still classified according to external clinical signs and this is mainly due to a lack in early biomarkers that could help to better identify subgroups of patients. What we hope having shown in this review is the high complexity of inflammatory events intermingled one to another, a complexity that may be extended more largely to all neurodegenerative diseases. Many studies revealed some inflammatory signs in ALS and FTD patients, animal models and also now in iPSC models. Nonetheless we still do not really understand if the inflammation is a driver or consequence of the pathology. Although some key aspects start to be lighted, questions remain: when exactly the inflammation is beneficial or detrimental? Which immune cells are responsible for the inflammatory responses? How the inflammatory events may differ between diverse genetic forms, especially in patients harboring mutations in genes known to be linked with immune functions?

A disappointing observation made today is that decades of clinical trials with immunomodulatory molecules have failed to prove their beneficial effects. Some explanations linked to pre-clinical protocols may explain these failures, such as the lack of power in numerous studies and the use of one single animal model that might not recapitulate all disease forms. Also, the timing of drug administration is often in early or pre-symptomatic stages in pre-clinical studies, a stage that cannot be targeted in enrolled patients. In this review we wanted also to focus on the tested immune modulatory drugs together with their unstudied potential targets that could impact the disease course. Indeed, most of the tested molecules target generic molecules, making difficult the monitoring of the real effects of the therapeutics. Our conclusion is that we still lack diagnostic tools and knowledge regarding the inflammatory events in ALS and FTD. More investigations are necessary before being able to design a treatment that could efficiently modulate inflammatory events. The multicellular implication is now widely accepted knowing that most genes are ubiquitously expressed. Thus, the use of a single drug targeting one pathway may be outdated. More personalized medicine begins to appear as a mean of integrating all factors of heterogeneity.

Until recently, ALS and FTD animal models allowed the study of genetic forms of the diseases. However, a lack of models recapitulating sporadic cases, remains. Additionally, the impossibility to have access to patient’s CNS cells and the rapidity of the neurodegenerative processes make difficult longitudinal studies with patients. In this context, iPSC offer unique opportunities to bypass these limitations. With the development of iPSC differentiation protocols to generate immune cells, researchers have now at their disposal tools to ask questions about interactions between ALS- or FDT-affected neurons and immune cells in a human context. These models may allow also to study a sequence of events. Of course, these protocols are still new and imperfect: cultures are not always pure, some cell subtypes can still not be generated and iPSCs are known to have a more embryonic than adult genomic memory (as opposed to transdifferentiated cells that do not go through an embryonic stage for their generation). This being, the current literature since several years has made the proof that it is possible to model diseases like ALS and FTD with iPS-derived neurons. In the recent years, several drugs have been tested and approved for clinical trials based on preclinical studies on iPS-derived neuronal cells (Fujimori et al., 2018; Okano et al., 2020). With regard to the innate and adaptive immune cells derived from iPSC, first papers showed that the generated cells were functional, more or less mature, and some functional intrinsic defects were shown, validating the use of these cells to identify further unknown mechanisms. To go one-step further, co-cultures are necessary to study interactions with neurons. Since few years, co-cultures, 3D cultures (e.g., microfluidic chips) as well as 3D brain and spinal organoids (Hor et al., 2018; Tan et al., 2021) are developed and available to study interactions between either MNs or cortical neurons affected mainly in ALS and FTD, respectively, and the various immune cells. Even if iPS-derived macrophages/microglia were the first generated immune cells, there is no report yet about iPSC modeling of ALS or FTD macrophage/microglia co-cultures with neurons. In their recent study, Pereira et al. (2021) describes the generation of human sensorimotor organoids with iPSC from ALS patients. They showed that these organoids contain microglia-like cells, but their study was not focused on the phenotypes of these cells, but rather on defects at the level of NMJs (Pereira et al., 2021). Another study describes the generation of cerebral organoids from iPSC of 3 FTD patients mutated in the tau gene (Bowles et al., 2021). Signs of neurodegeneration, fewer excitatory neurons and elevated levels of inflammation were observed in 6-month old cerebral organoids, showing that it was possible to recreate much of the damage seen in FTD. Moreover, they were able to prevent neuron death with an experimental drug. Even if microglial cells were not studied directly in these organoids, an inflammatory response was observed suggesting that this pathological feature could be modeled in such 3D models. Moreover, it was recently shown that exposure of brain models to serum mimics age associated BBB breakdown (Chen et al., 2021), providing a platform to look for effective treatments for disorders with an age component like ALS and FTD.

As a final word, the iPSC technology grants new prospects to better understand and model inflammatory events in different FTD and ALS disease forms. Co-cultures are offering a new era of researches with more integrated models which we hope will lead to new ways to perform drug screening and offer adapted therapeutic opportunities for ALS and FTD patients.

## Author Contributions

All authors listed have made a substantial, direct, and intellectual contribution to the work, and approved it for publication.

## Conflict of Interest

The authors declare that the research was conducted in the absence of any commercial or financial relationships that could be construed as a potential conflict of interest.

## Publisher’s Note

All claims expressed in this article are solely those of the authors and do not necessarily represent those of their affiliated organizations, or those of the publisher, the editors and the reviewers. Any product that may be evaluated in this article, or claim that may be made by its manufacturer, is not guaranteed or endorsed by the publisher.

## Footnotes

1
https://clinicaltrials.gov
